# Deciphering the Magnetostructural Criteria in Dy^III^ and Ho^III^ Macrocycle-Based Single-Molecule Magnets with Pseudo‑*D*_5h_ Symmetry: A Combined Single-Crystal Hysteresis and Theoretical Study

**DOI:** 10.1021/acs.inorgchem.5c00644

**Published:** 2025-06-05

**Authors:** Alexandros S. Armenis, Georgia P. Bakali, Sagar Paul, Konstantinos N. Pantelis, Luís Cunha-Silva, Jinkui Tang, Dimitris I. Alexandropoulos, Wolfgang Wernsdorfer, Eufemio Moreno-Pineda, Theocharis C. Stamatatos

**Affiliations:** † Department of Chemistry, 37795 University of Patras, Patras 265 04, Greece; ‡ Physikalisches Institut, 150232 Karlsruhe Institute of Technology (KIT), Kaiserstraße 12, Karlsruhe D-76131, Germany; § LAQV/REQUIMTE & Department of Chemistry and Biochemistry, Faculty of Sciences, University of Porto, Porto 4169-007, Portugal; ∥ State Key Laboratory of Rare Earth Resource Utilization, 58277 Changchun Institute of Applied Chemistry, Chinese Academy of Sciences, Changchun 130022, P.R. China; ⊥ Institute for Quantum Materials and Technology (IQMT), 150232 Karlsruhe Institute of Technology (KIT), Hermann-von-Helmholtz-Platz 1, Eggenstein-Leopoldshafen D-76344, Germany; # Facultad de Ciencias Naturales, Exactas y Tecnología, Depto. de Química-Física, Universidad de Panamá, Panamá 0824, Panama; ∇ Facultad de Ciencias Naturales, Exactas y Tecnología, Grupo de Investigación de Materiales, Universidad de Panamá, Panamá 0824, Panama; ○ Foundation for Research and Technology-Hellas (FORTH/ICE-HT), Institute of Chemical Engineering Sciences, Platani, P.O. Box 1414, Patras 26504, Greece

## Abstract

The employment of the metal-ion-assisted [1+1] Schiff-base condensation has led to complexes [Ln­(L^N5^)­(Ph_3_SiO)_2_]­(PF_6_), where Ln^III^ = Dy^III^ (**1-Dy**) and Ho^III^ (**1-Ho**), with close-to-ideal *D*_5h_ local symmetry. For the Kramers Dy^III^ system, slow magnetization relaxation was observed up to 64 K yielding a sizable *U*_eff_ value of 963 K, whereas the non-Kramers Ho^III^ compound exhibited no out-of-phase signals at *T* > 2 K. Single-crystal magnetic hysteresis measurements uncovered the magnetization blockage of **1-Dy** at *T* < 4 K with typical butterfly shaped loops, while open rectangular-shaped hysteresis loops were observed for **1-Ho** below 0.2 K. Doping of the **1-Ho** compound into a diamagnetic Y^III^ matrix unveiled the hyperfine-driven QTM steps reflected by staircase-like hysteresis loops for **1-Ho@Y**. Detailed analysis through ab initio calculations has shed light on the low-lying energy levels of both compounds leading to well-isolated and pure ground states of *m*_*J*_ = ±15/2 and *m*_*J*_ = ±8 for **1-Dy** and **1-Ho**, respectively. The magnetization relaxation for **1-Dy** advances through the third excited state, whereas the significant tunnel splitting (Δ_tun_) of ∼0.15 cm^–1^ in the ground state of **1-Ho** has fostered the onset of fast tunneling relaxation.

## Graphical abstract

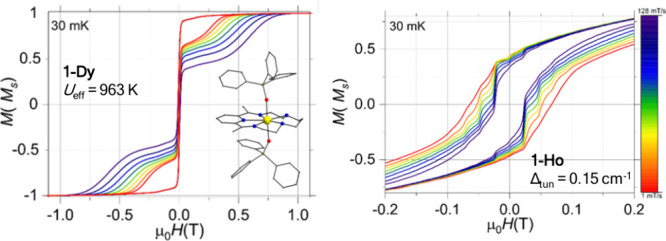

## 1 Introduction

For more than 30 years, a class of coordination compounds that exhibit interesting magnetic and quantum phenomena of purely molecular origin are dubbed Single-Molecule Magnets (SMMs), and they have been a hot topic within the field of Molecular Magnetism.^1^ These complex magnetic entities manifest slow relaxation of their magnetization due to an anisotropic effective energy barrier (*U*_eff_) for the reversal of their magnetic moment at low temperatures, and superparamagnetic blocking below a critical temperature coined as *T*_B_.^2^ Interestingly, the rich quantum mechanical properties of these molecules, such as quantum tunneling of magnetization,^3^ quantum phase interference,^4^ and quantum coherence,^5^ postulate them as building units for high-density storage devices and quantum information processing acting as quantum bits.^6^

Since Ishikawa and co-workers groundbreaking discovery of slow magnetization relaxation in a mononuclear coordination compound with the general formula [LnPc]^−^ (Ln^III^ = Tb^III^, Dy^III^), a new class of SMMs has emerged, known as mononuclear SMMs or Single-Ion Magnets (SIMs).^7^ These compounds feature an anisotropic lanthanide ion positioned between two phthalocyanine (Pc^–^) ligands, marking a significant advancement in the field. Specifically, in the Tb^III^ complex, the *D*_4d_ crystal field around the metal center stabilized an *m*_*J*_ = ±6 ground state, separated by ∼600 K from the first excited state, creating an unprecedented energy barrier for magnetization reversal. The anisotropy originates from the strong intrinsic spin–orbit coupling of lanthanides, yielding highly anisotropic ground states with large magnetic moments.^8^ Numerous mononuclear lanthanide complexes have since been reported with enhanced SMM properties, exhibiting high *U*_eff_ values and blocking temperatures.^9^ The magnetic performance depends on the oblate/prolate nature of the lanthanide ions, along with the symmetry and strength of the ligand field.^10^ Additionally, magnetic bistability is influenced by time-reversal symmetry according to Kramers theorem, where lanthanides with half-integer *J* values (Kramers ions) maintain double degeneracy, while integer *J* values (non-Kramers ions) require highly symmetric ligand fields to exhibit enhanced SMM properties.^11^

Kramers ions, such as Dy^III^ and Er^III^, achieve high energy barriers for spin reversal when placed in specific local metal symmetries and ligand fields that stabilize the highest ±*m*_*J*_ values.^12^ For the oblate Dy^III^ ion, hexagonal bipyramidal (*D*_6h_) coordination geometries with strong axial crystal fields have led to record anisotropy barriers exceeding 2400 K, and blocking temperatures up to 30 K.^13^ On the other hand, non-Kramers ions, such as Tb^III^ and Ho^III^, possess a doubly degenerate ground state and demonstrate SMM behavior when only subjected to highly symmetric and axial ligand fields, due to the easily mixed *m*_*J*_ states through perturbations, such as transverse crystal field anisotropy, intermolecular magnetic interactions, and interactions with nuclear magnetic spins.^12,14^ Along these lines, the highest reported energy barriers for Tb^III^ have been achieved under a *D*_4d_ symmetry,^15^ whereas for Ho^III^ a molecular *D*_5h_ symmetry has been proved to be an effective route to significant *U*_eff_ values.^16^

Several pentagonal bipyramidal Dy^III^ mononuclear complexes have been reported as exceptional SMMs, exhibiting high magnetization reversal barriers and blocking temperatures, since the mixing of the magnetic states from crystal field terms can be greatly reduced, minimizing the tunneling relaxation.^12,17^ Notably, the compounds [Dy­(Cy_3_PO)_2_(H_2_O)_5_]­X_3_ (X^–^ = Cl^–^ or Br^–^)^18^ and [Dy­(O^*t*^Bu)_2_(py)_5_]­(BPh_4_)^19^ have gained significant attention for their outstanding magnetic performance. These complexes exhibit near-ideal *D*_5h_ symmetry, with the Dy^III^ centers coordinated equatorially by five neutral monodentate ligands and axially by two strong O-donor ligands. Hence, this symmetric environment proved to be an ideal testbed for non-Kramers and oblate-shaped Ho^III^ SMMs. In 2017, Tong and co-workers reported the first Ho^III^ pentagonal bipyramidal SMM, [Ho­(CyPh_2_PO)_2_(H_2_O)_5_]­I_3_, achieving a *U*_eff_ of 340 K.^20^ In 2020, Zheng et al. replaced equatorial aqua ligands with pyridines and introduced axial trimethylsiloxides, boosting the energy barrier to a record value of 715 K for Ho^III^ SMMs.^21^ Although the fascinating SMM behavior of these molecular systems offers new perspectives for synthetic chemists, pentagonal bipyramidal Ho^III^ complexes remain largely unexplored with only a handful of examples reported in the literature.^22^ This is obviously due to the challenging part of this synthetic strategy, i.e., to achieve control and chemically drive the binding of the monodentate soft-groups at the equatorial positions, while leaving the apical sites for the strongly bound O-donor ligands.

In contrast, Schiff-base macrocyclic ligands provide an easy and controllable way to manipulate the crystal field local symmetry around an oblate lanthanide ion.^23^ These ligands encapsulate the metal center with five or six equatorial soft donor atoms, while bulky and strong axial O-donor ligands, such as phenoxides or siloxides, enhance axial magnetic anisotropy, thus stabilizing *D*_5h_ or *D*_6h_ symmetries, respectively.^23^ The macrocycle approach, which is based on the enhanced thermodynamic stability imposed by the macrocycle effect, was first introduced by Murrie and co-workers in 2019 through the employment of the [1+1] and [2+2] Schiff-base condensation reactions toward the isolation of pentagonal and hexagonal bipyramidal Dy^III^ SMMs, respectively.^24^ The lanthanide ion is surrounded by an N_5_ or N_6_ macrocycle, resulting in pentagonal or hexagonal equatorial planes, and the axial sites are occupied by anionic triphenylsiloxide ligands, thus leading to high axial anisotropy and *U*_eff_ values exceeding 1000 K. Over the past 5 years several macrocyclic Dy^III^ complexes have been reported featuring pentagonal or hexagonal bipyramidal symmetries and exhibiting high-performance SMM properties.^25^ However, the macrocycle approach has not been applied so far toward the isolation of mononuclear Ho^III^ complexes, and their SMM behavior is practically unexplored, especially in comparison to their Dy^III^ counterparts.

Herein, we report the first comparative study of the isostructural mononuclear lanthanide macrocyclic complexes with the general formulas [Ln­(L^N5^)­(Ph_3_SiO)_2_]­(PF_6_), where Ln^III^ = Dy^III^ (**1-Dy**) and Ho^III^ (**1-Ho**), in which the lanthanide ions are surrounded by a pentadentate N-donor macrocyclic ligand (L^N5^) resulting from a metal-ion assisted [1+1] condensation reaction. The sterically demanding axial triphenylsiloxide (Ph_3_SiO^–^) groups along with the planar conformation of the L^N5^ macrocycle have led to *D*_5h_ local symmetry around the metal centers. Therefore, the enhanced axial anisotropy, provided by the apical ligands, afforded out-of-phase (χ_M_″) signals up to 64 K and a high magnetization reversal barrier of 963 K for the Dy^III^ complex, whereas the Ho^III^ (and the doped Ho@Y) analogue surprisingly did not exhibit χ_M_″ signals at *T* > 2 K, either in the absence or presence of an external dc field. The combined analysis of hysteresis loops (through single-crystal studies) and the low energy electronic spectrum (through ab initio calculations) of the Ho^III^ complex revealed a highly anisotropic *m*_*J*_ = ±8 ground state well separated (∼429 K) from the first excited one, but the presence of a large tunneling gap (Δ_tun_ = 0.15 cm^–1^) within the ground quasi-doublet has boosted fast tunneling relaxation between the lowest *m*_*J*_ = ±8 substates.

## 2 Experimental Section

### 2.1 Synthesis

Aerobic conditions were applied to all manipulations using materials (reagent grade) and solvents as received. No uncommon hazards were noted.

#### 2.1.1 [Dy­(L^N5^)­(Ph_3_SiO)_2_]­(PF_6_) (1-Dy)

2,6-Diacetylpyridine (0.10 mmol, 0.016 g), *N*1,*N*1′-(propane-1,3-diyl)­bis­(ethane-1,2-diamine) (0.10 mmol, 0.017 mL), and Dy­(NO_3_)_3_·5H_2_O (0.10 mmol, 0.044 g) were transferred to a round-bottom flask containing 10 mL of MeOH resulting in a pale yellow clear solution. The mixture was refluxed under magnetic stirring for 24 h leading to a bright yellow clear solution, and then the solvent was removed under reduced pressure yielding a yellow oil. To the oily residue was added a CH_2_Cl_2_ solution (10 mL) containing Ph_3_SiOH (0.20 mmol, 0.111 g) and NEt_3_ (0.20 mmol, 0.028 mL), and subsequently an aqueous solution (10 mL) of KPF_6_ (0.10 mmol, 0.018 g), thus creating two layers (organic and aqueous phases). The reaction mixture was refluxed for an hour, the yellow CH_2_Cl_2_ phase was separated with a separating funnel and then filtered, giving a pale yellow clear solution. Colorless plate-like crystals suitable for single-crystal X-ray diffraction were isolated after 2–3 days by layering the CH_2_Cl_2_ solution with *n*-hexanes (10 mL). The crystals were collected by filtration, washed with CH_2_Cl_2_ (2 × 2 mL), and dried in air. The yield was 42% (based on Dy). The air-dried microcrystalline solid was analyzed as **1-Dy**. Anal. calcd for C_52_H_55_DyF_6_N_5_O_2_PSi_2_: C, 54.53; H, 4.84; N, 6.11%. Found: C, 54.62; H, 4.90; N, 6.04%.

#### 2.1.2 [Ho­(L^N5^)­(Ph_3_SiO)_2_]­(PF_6_) (1-Ho) and [Y­(L^N5^)­(Ph_3_SiO)_2_]­(PF_6_) (1-Y)

The isostructural complex **1-Ho** and the structurally similar compound **1-Y** were prepared in crystalline forms using the same synthetic protocol as that of **1-Dy** through the replacement of Dy­(NO_3_)_3_·5H_2_O with Ho­(NO_3_)_3_·5H_2_O (0.10 mmol, 0.044 g) and Y­(NO_3_)_3_·6H_2_O (0.10 mmol, 0.038 g), respectively. The yields were 46% for **1-Ho** (based on Ho) and 48% for **1-Y** (based on Y). The air-dried microcrystalline solids were analyzed as **1-Ho** and **1-Y**. Anal. calcd for C_52_H_55_HoF_6_N_5_O_2_PSi_2_: C, 54.40; H, 4.83; N, 6.10%. Found: C, 54.51; H, 4.91; N, 6.03%. Anal. calcd for C_52_H_55_YF_6_N_5_O_2_PSi_2_: C, 58.26; H, 5.17; N, 6.53%. Found: C, 58.12; H, 5.06; N, 6.61%. The diluted sample **1-Ho@Y** was synthesized following the same protocol as of the pure compounds, but using a Ho­(NO_3_)_3_·5H_2_O:Y­(NO_3_)_3_·6H_2_O ratio of 1:19. The identity of crystalline **1-Ho@Y** was further confirmed by unit cell comparison with the cell parameters of pure **1-Y**, thus confirming its isostructural nature with **1-Y**.

### 2.2 X-ray Crystallography

Colorless block-like crystals of **1-Dy** were collected from the crystallization vial, immediately immersed in a highly viscous oil, and a suitable crystal (0.19 × 0.11 × 0.07 mm) was mounted on a CryoLoop.^26^ Diffraction data were collected on a Bruker D8 diffractometer (Mo Kα graphite-monochromated radiation, λ = 0.71073 Å) with the acquisition controlled by the APEX2 software package.^27^ The temperature of acquisition was 150(2) K, and it was set up with a cryosystem by the Oxford Cryosystems Series 700. Images were processed with the software SAINT+,^28^ and absorption effects were corrected with the multiscan method implemented in SADABS.^29^ The structure was solved using SHELXTL incorporated in the Bruker APEX-III software package and refined using the SHELXLE.^26,30^ All the non-hydrogen atoms of the structure were successfully refined using anisotropic displacement parameters. H-atoms bound to C-atoms were placed at geometrical positions using the suitable *HFIX* instructions in SHELXL and included in subsequent refinement cycles in riding-motion approximation with isotropic thermal displacement parameters (*U*_iso_) fixed at the carbon atom to which they are attached.

Data for compounds **1-Ho** and **1-Y** were collected on a Rigaku XtaLAB Synergy-S single-crystal X-ray diffractometer equipped with a CCD area detector and a graphite monochromator utilizing Cu Kα radiation (λ = 1.54184 Å). Selected block-like colorless crystals of **1-Ho** (0.30 × 0.19 × 0.13 mm) and **1-Y** (0.276 × 0.162 × 0.157 mm) were attached to glass fiber with paratone-N oil and transferred to a goniostat for data collection. Empirical absorption corrections (multiscan based on symmetry-related measurements) were applied using CrysAlis RED software.^31^ The structures were solved by direct methods using SIR92,^32^ and refined on *F* ^2^ using SHELXL97,^33^ SHELXL-2014/7,^34^ and SHELXT.^35^ Software packages used: CrysAlisCCD^31^ for data collection, CrysAlisRED^31^ for cell refinement and data reduction, and WINGX for geometric calculations.^36^ For both compounds **1-Ho** and **1-Y**, all the non-H-atoms were treated anisotropically, whereas the H-atoms were placed in calculated, ideal positions and refined as riding on their respective C-atoms. Various figures of all the structures were created using Mercury^37^ and Diamond^38^ software packages.

### 2.3 Physical Measurements

Elemental analyses (C, H, and N) were performed by the University of Patras microanalytical service. Infrared (IR) spectra (4000–400 cm^–1^) were recorded in the solid state using a PerkinElmer 16 PC spectrometer with samples prepared as KBr pellets (Figures S1–S3). Powder-XRD measurements were carried out on a Rigaku Miniflex 6G X-ray Diffractometer. Magnetic susceptibility studies were performed in the temperature range 1.9–300 K using a Quantum Design MPMS XL-7 SQUID magnetometer equipped with a 7 T magnet. The direct current (dc) magnetic susceptibility measurements were performed with an external magnetic field of 1000 Oe in the temperature range 1.9–300 K, and the alternating current (ac) measurements were measured in a 3.0 Oe ac field oscillating at different frequencies from 1 to 1000 Hz. The experimental magnetic susceptibility data were corrected for the diamagnetism estimated from Pascal’s tables and sample holder calibration.^39^ Low-temperature (30 mK–5.0 K) magnetization (*M*) versus field (*H*) measurements were performed on a single crystal using an array of μ-SQUIDs inside a dilution refrigerator equipped with a 3D vector magnet.^40^ The high sensitivity of this magnetometer allows the study of single crystals of SMMs of the order 10–500 μm. The field can be applied in any direction with a precision better than 0.1° by separately driving three orthogonal coils. Crystals of **1-Dy**, **1-Ho**, and **1-Ho@Y** were maintained in mother liquor to avoid degradation and were covered in Apiezon grease for protection during the transfer to the μ-SQUID and thermalization during subsequent cooling.

### 2.4 Computational Studies

Ab initio calculations employing the CASSCF/SO-RASSI/SINGLE_ANISO approach implemented in the OpenMolcas package^41^ were carried out for compounds **1-Dy** and **1-Ho**. For the calculations, the crystal structures were employed without further optimizations and the atoms were described with the standard basis sets from the ANO-RCC library.^42^ A basis set of VTZP quality was employed for the Dy­(III) and Ho­(III) ions, while VDZP quality was employed for atoms directly bound to the Ln­(III) ions, and VDZ quality for the remaining atoms, using the second-order DKH transformation.^43^ Optimisation of the molecular orbitals (MOs) was achieved by state-averaged CASSCF calculations. For Dy­(III), the active space was defined by the nine 4f electrons in the seven 4f orbitals, while for Ho­(III) the active space corresponded to ten 4f electrons in 4f orbitals. For the Dy-analogue, three calculations were carried out (RASSCF routine) with 21, 224, and 490 states for *S* = 5/2, *S* = 3/2 and *S* = 1/2, respectively. The CASSCF wave functions were subsequently mixed by spin–orbit coupling, employing the RASSI routine^44^ with all 21 states for *S* = 5/2 being included, while 128 and 130 states were included for *S* = 3/2 and *S* = 1/2. Lastly, the crystal field decomposition of the ground *J* = 15/2 multiplet of the ^6^H_15/2_ term was executed with the SINGLE_ANISO module. For the Ho-analogue, three calculations were carried out (RASSCF routine) with 35, 210, and 196 states for *S* = 2, *S* = 1 and *S* = 0, respectively. The CASSCF wave functions were subsequently mixed by spin–orbit coupling, employing the RASSI routine with all 35 states for *S* = 2 being included, while 117 and 75 states were included for *S* = 2 and *S* = 0. Lastly, the crystal field decomposition of the ground *J* = 8 multiplet of the ^5^I_8_ term was executed with the SINGLE_ANISO module.^44b,44c^

## 3 Results and Discussion

### 3.1 Synthetic Comments

Our research group has recently reported the syntheses of some new [1+1] Schiff-base macrocyclic scaffolds for the isolation of hexagonal bipyramidal Dy^III^ complexes with high-performance SMM properties.^25m,25n^ Interestingly, the [1+1] Schiff-base condensation approach has been proven also a successful synthetic route toward pentagonal bipyramidal Dy^III^ complexes featuring enhanced axial anisotropy and large energy barriers for the magnetization reversal.^24b,25d,25k,25l^ Thus, the macrocycle approach appears to be a fruitful route for the enrichment of the axiality of oblate-shaped anisotropic lanthanide ions such as Dy^III^ and Ho^III^, since the soft equatorial ligation minimizes the electrostatic repulsion between the 4f electron density and the donor atoms. In this line, we synthesized the mononuclear Dy^III^ and Ho^III^ complexes bearing the [1+1] Schiff base macrocycle L^N5^, implementing an established synthetic protocol (Scheme 1). In particular, the stoichiometric reaction of 2,6-diacetylpyridine and the tetramine *N*1,*N*1′-(propane-1,3-diyl)­bis­(ethane-1,2-diamine) was carried out in the presence of Ln­(NO_3_)_3_·5H_2_O, as a templating agent to form in situ the “Ln­(L^N5^)­(NO_3_)_3_” precursor. Subsequently, the bulky triphenylsilanol ligand (Ph_3_SiOH) was used, which upon deprotonation with NEt_3_ can occupy the axial sites of the Ln^III^ center and displace the coordinated NO_3_^–^ ions and/or solvate molecules (MeOH, H_2_O). Moreover, the KPF_6_ salt was chosen as a means of supplying the reaction with PF_6_^–^ ions which are excellent (high symmetry and bulky) counterions for mononuclear Ln^III^ macrocyclic complexes. Finally, the employed solvent mixture (CH_2_Cl_2_/H_2_O) ensures that the Ln^III^ macrocyclic complex dissolves in the organic phase, while the byproducts, KNO_3_ and (Et_3_NH)­(NO_3_), are transferred into the aqueous phase. Accordingly, separation and layering of the CH_2_Cl_2_ phase with *n*-hexanes to aid crystallization has afforded colorless plate-like crystals of the targeted complexes [Dy­(L^N5^)­(Ph_3_SiO)_2_]­(PF_6_) (**1-Dy**) and [Ho­(L^N5^)­(Ph_3_SiO)_2_]­(PF_6_) (**1-Ho**). The selection of 2,6-diacetylpyridine as the ‘head’ unit of the L^N5^ macrocycle was supported by some previously reported mononuclear Dy^III^ macrocyclic complexes with different polyamines, pseudo-*D*_5h_ symmetry and enhanced SMM properties (Table S1). Notably, **1-Ho** is the first structurally characterized compound derived from the [1+1] Schiff-base condensation approach and exhibiting SMM behavior under a pentagonal bipyramidal pseudosymmetry.

**Scheme 1:**
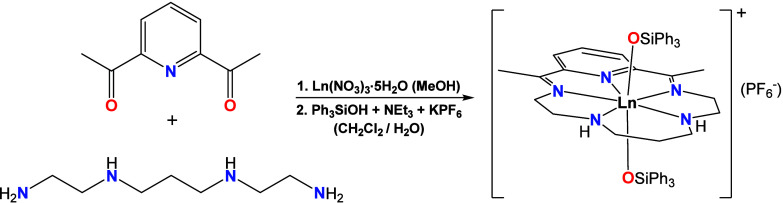
General Synthetic Route for the Preparation of the Pentagonal Bipyramidal Ln^III^ (Ln = Dy, Ho) Macrocyclic Complexes Reported in This Study

### 3.2 Description of Structures

Single-crystal X-ray diffraction studies revealed the structures of molecular complexes **1-Dy** and **1-Ho**, which both crystallize in the monoclinic space group *P*2_1_/*c*, whereas **1-Y** crystallizes in monoclinic *C*2/*c*. Crystallographic details for all compounds are presented in Table S2. Selected interatomic distances and angles for complexes **1-Dy** and **1-Ho** are listed in Tables S3 and S4. Figures 1A and 2A illustrate the mononuclear cationic compounds [Dy­(L^N5^)­(Ph_3_SiO)_2_]^+^ for **1-Dy** and [Ho­(L^N5^)­(Ph_3_SiO)_2_]^+^ for **1-Ho**, respectively, each counterbalanced by one PF_6_^–^ ion. In both compounds, the lanthanide metal center resides in the cavity of the equatorial N-donor (N_eq_) macrocyclic ligand (L^N5^), while the apical positions are occupied by the triphenylsiloxide (Ph_3_SiO^–^) groups (O_ax_). Each Ln^III^ ion is coordinated equatorially to one pyridine N atom, two imine N atoms and two N atoms belonging to the secondary amine (>NH−) groups, while two O-donor atoms of the siloxide ligands are axially coordinated, leading to an overall N_5_O_2_ coordination environment. In both compounds, the N_eq_-Ln-N_eq_ angles range from 65.8(1)° to 87.0(1)° and the O_ax_–Ln–N_eq_ angles vary between 87.0(9)–94.4(8)°, which deviate more pronounced or slightly from the ideal 72° and 90° of a pentagonal bipyramidal geometry, respectively (Tables S3 and S4). In addition, the almost linear O_ax_–Dy–O_ax_ and O_ax_–Ho–O_ax_ angles of 175.6(1)° for **1-Dy** and 174.8(8)° for **1-Ho**, respectively, lead to a moderately distorted pentagonal bipyramidal coordination geometry around the metal centers according to the SHAPE program (Table S5, Figures 1B, and 2B).^45^ Interestingly, both complexes exhibit lower Continuous Shape Measure (CShM) values for *D*_5h_ symmetry (∼1.07) than the similarly reported compound [Dy­(L^N5^)­(Ph_3_SiO)_2_]­(BPh_4_) with CShM = 1.24–1.39 (Table S1),^25k^ reinforcing the influence of the bulkier BPh_4_^–^ counterion on the deviation from the ideal polyhedron. Complex [Dy­(L^N5^)­(Ph_3_SiO)_2_]­(BPh_4_) consists of two crystallographically independent monomeric Dy^III^ complexes within the unit cell, each of them manifesting different distortions from the ideal pentagonal bipyramidal geometry. More specifically, the O_ax_–Dy–O_ax_ bond angles are 173.33(16)° and 170.78(16)°, slightly more bent than that of **1-Dy**, whereas the Dy–O_ax_ bond lengths of 2.150(4)/2.173(4) and 2.157(4)/2.159(9) Å are of similar strength with **1-Dy** (Figure 1C), while the Dy–N_eq_ bond lengths are nearly identical in both complexes.

**Figure 1:**
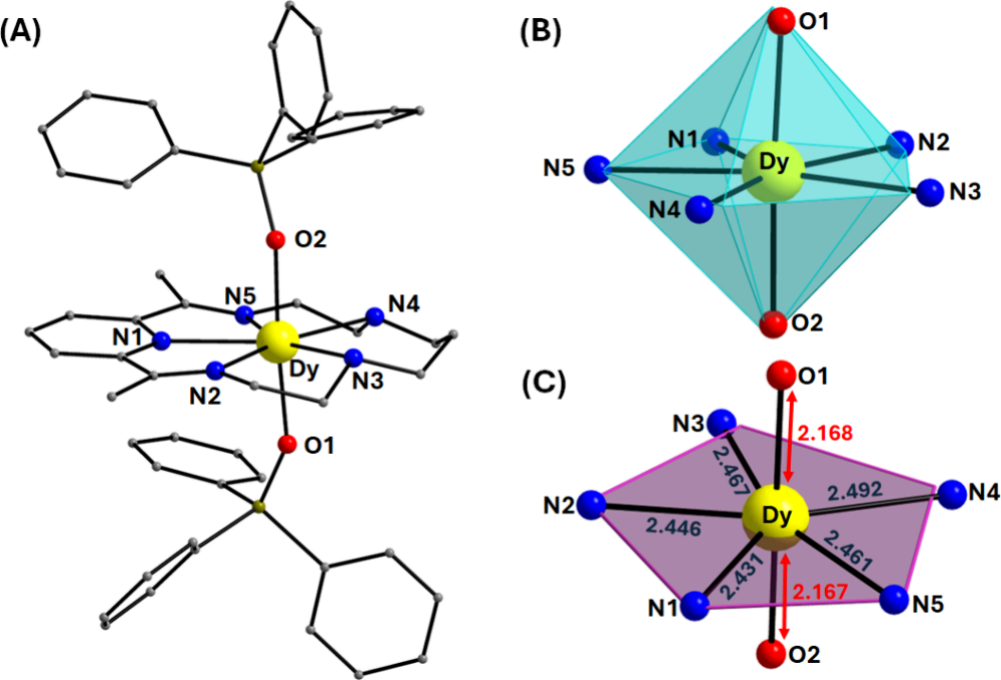
(A) Labeled representation of the cationic complex of **1-Dy**. (B) Pentagonal bipyramidal coordination polyhedron of the Dy^III^ center. (C) Deviation of the N_eq_-atoms from the ideal pentagonal plane along with the Dy–O_ax_/N_eq_ bond distances (Å). The PF_6_^–^ counterions, and the H atoms are omitted for clarity. The smaller white spheres define the vertices of the corresponding ideal polyhedron. Color scheme: Dy, yellow; O, red; N, blue; C, gray; Si, olive.

**Figure 2:**
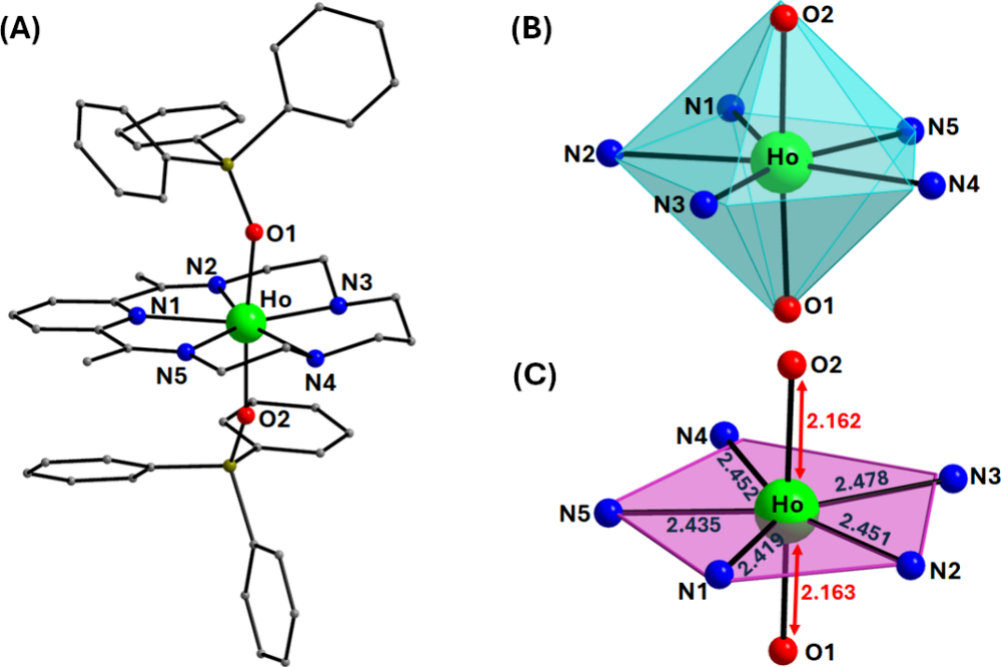
(A) Labeled representation of the cationic complex of **1-Ho**. (B) Pentagonal bipyramidal coordination polyhedron of the Ho^III^ center. (C) Deviation of the N_eq_-atoms from the ideal pentagonal plane along with the Ho–O_ax_/N_eq_ bond distances (Å). The PF_6_^–^ counterions, and the H atoms are omitted for clarity. The smaller white spheres define the vertices of the corresponding ideal polyhedron. Color scheme: Ho, green; O, red; N, blue; C, gray; Si, olive.

Furthermore, the deviation of the N_eq_-atoms in **1-Dy** from the ideal pentagonal plane, which is depicted with purple in Figures 1C and 2C, has resulted in very low CShM values of 0.79 (**1-Dy**) and 0.75 (**1-Ho**), indicating the overall planarity of the macrocyclic L^N5^ ligand. The compressed nature of the pentagonal bipyramidal coordination polyhedron of the metal centers stems from the short, axial Dy/Ho–O_ax_ bonds in contrast to the longer Dy/Ho–N_eq_ bond lengths in the equatorial plane, thus favoring the axial crystal field required for oblate shaped Ln^III^ ions. Additionally, the packing diagram of neighboring monomeric complexes revealed that the shortest intermolecular Ln···Ln distances are 10.394(4) Å for **1-Dy** and 10.402(5) Å for **1-Ho** (Figures S4 and S5).

### 3.3 Magnetic Studies

Direct current (dc) magnetic susceptibility measurements were carried out on microcrystalline samples of analytically pure complexes **1-Dy** and **1-Ho**, as derived from elemental analysis studies, in the 2–300 K range under an applied magnetic field of 1000 Oe (Figures 3A and 3C). In addition, the powder X-ray diffraction patterns of all compounds show good agreement with the simulated ones, confirming the phase purity of the samples (Figures S6–S8). The observed room-temperature χ_Μ_ *Τ* values of 15.50 (**1-Dy**) and 12.80 cm^3^ mol^–1^ K (**1-Ho**) are close to the theoretical ones of 14.17 cm^3^ mol^–1^ K for an isolated Dy^III^ Kramers ion (^6^H_15/2_, *S* = 5/2, *L* = 5, *g* = 4/3), and 14.07 cm^3^ mol^–1^ K for an isolated Ho^III^ non-Kramers ion (^5^I_8_, *S* = 2, *L* = 6, *g* = 5/4). The χ_Μ_ *Τ* product smoothly decreases upon lowering the temperature until 7 K, and then more sharply reaching a value of 5.40 cm^3^ mol^–1^ K at 2 K for **1-Dy**, whereas for compound **1-Ho** there is a steady decline of the χ_Μ_ *Τ* product across the entire temperature range leading to a value of 10.90 cm^3^ mol^–1^ K at 2 K. Such a steep decrease of the χ_Μ_ *Τ* indicates the onset of magnetic blocking, where pinning of the magnetic moment in the immobilized crystalline material occurs. That is, below 7 K, the magnetic moments of individual crystallites in the sample are pinned along a preferred axis and do not respond to an external magnetic field, which is a common phenomenon in SMMs with large energy barriers. The isothermal field (*H*) dependence of the magnetization (*M*) was measured at 2, 5, and 7 K for **1-Dy**, and at 3 and 5 K for **1-Ho** in the field range of 0–7 T (Figure 3B,D). The *M*(*H*) curves at 2 K are similar for both complexes, exhibiting a rapid increase of magnetization at low fields while remaining almost constant at high fields. The magnetization values at the maximum applied field of 7 T and the lowest temperature of 2 K are 5.8 (**1-Dy**) and 5.9 *Ν*_A_μ_Β_ (**1-Ho**), much lower than the expected saturation value (*M*_S_) for one free Dy^III^ or Ho^III^ ion (*M*_S_/*N*_A_μ_Β_ = 10*N*_A_μ_Β_); this can be ascribed to the crystal field effects that induce significant magnetic anisotropy. On measuring the temperature dependence of the magnetization under zero-field cooled (ZFC) and field-cooled conditions we can extract the blocking temperature (*T*_B_), which corresponds to the maximum point of the ZFC magnetization.^46^ Hence, as shown in Figure S9, it becomes apparent that for complex **1-Dy** the magnetization is blocked at a temperature of ∼4 K.

**Figure 3:**
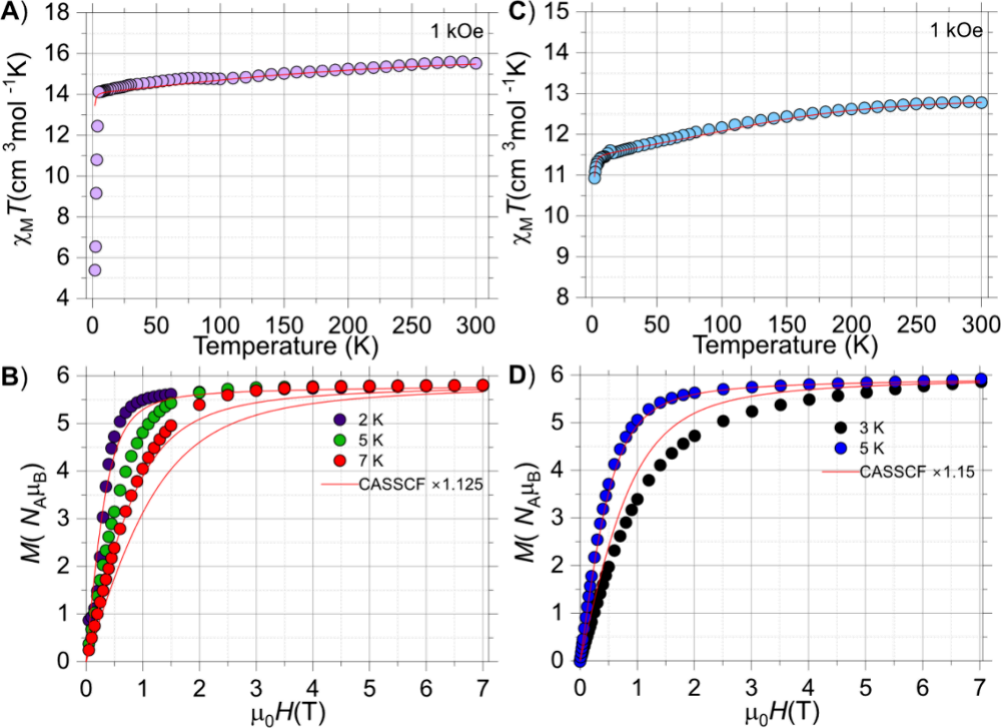
Temperature dependence of the χ_Μ_ *Τ* product for complexes **1-Dy** (A) and **1-Ho** (C) at an applied field of 1000 Oe. Magnetization (*M*) vs field (*H*) plots at various low temperatures for complexes **1-Dy** (B) and **1-Ho** (D). Red solid lines were obtained by employing the crystal field parameters from CASSCF calculations (see ESI for details).

To investigate the magnetic relaxation properties of complex **1-Dy**, alternating current (ac) magnetic susceptibility measurements were conducted at a zero applied dc field under a weak ac field of 3.0 G oscillating at frequencies of 1–1000 Hz. Interestingly, compound **1-Dy** exhibits frequency- and temperature-dependent in-phase (χ_Μ_′) and out-of-phase (χ_Μ_″) magnetic susceptibility signals in the 1.9–80 K region, with well-defined peak maxima up to ∼64 K (Figure 4). The appearance of out-of-phase peaks of signals denotes the presence of slow magnetization relaxation via an energy barrier for the spin-reversal, which is consistent with an efficient SMM behavior. At temperatures below ∼10 K, only tails of signals in the χ_M_″ vs *T* plots were detected, which are suggestive of the onset of quantum tunneling of magnetization (QTM) relaxation process (Figure S10). At elevated temperatures, the maxima become significantly dependent on temperature, shifting to higher frequencies as the temperature increases, indicating a relaxation process driven by thermal activation.^47^

**Figure 4:**
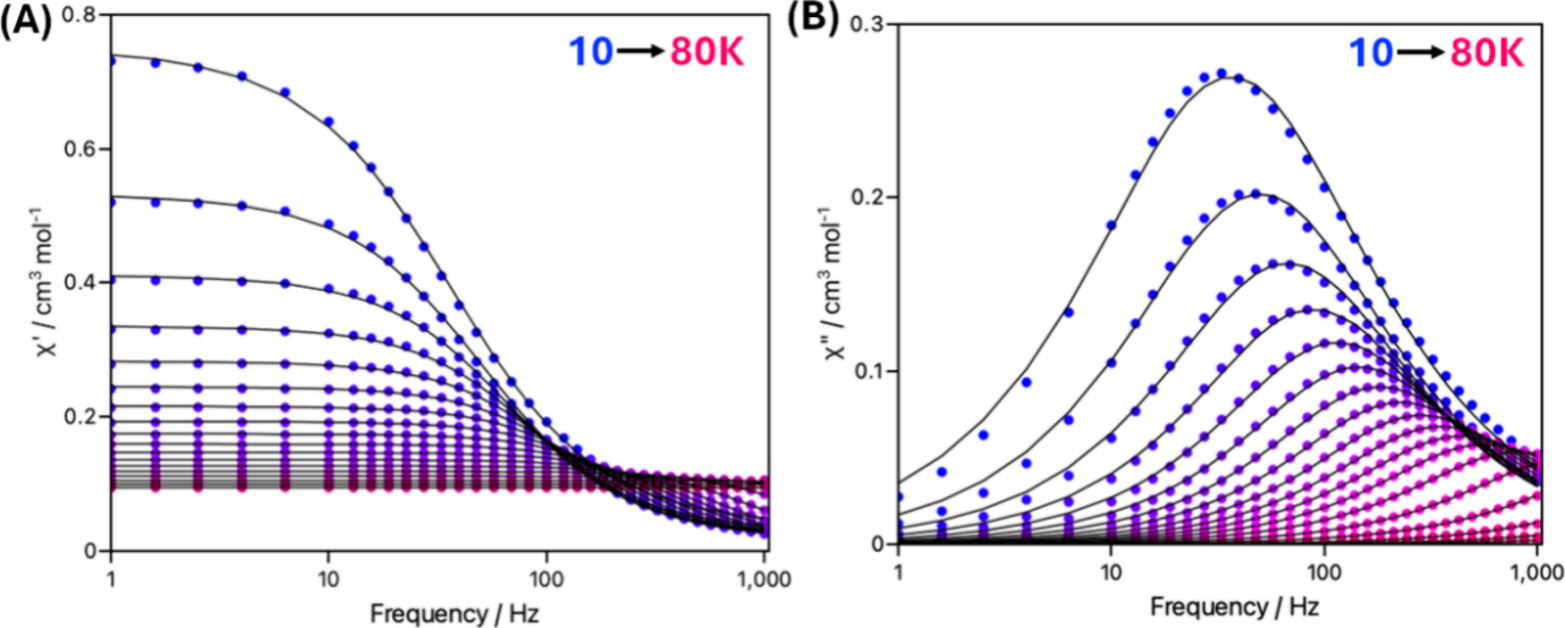
Frequency-dependence of the in-phase (A) and out-of-phase (B) magnetic susceptibilities under zero applied dc field over the temperature range of 10–80 K for complex **1-Dy**. The solid lines are the best-fit of the data.

Therefore, to deduce the temperature-dependence of relaxation times (τ) and assess the operating relaxation processes, we constructed the Arrhenius plot (lnτ vs 1/*T*) and the data were fitted according to the eq 1:

$$\tau^{-1}={\tau_{0}}^{-1}e^{-U\mathrm{eff}/kT}+CT^{n}+{\tau_{\mathrm{QTM}}}^{-1}$$ 1

where the pre-exponential factor, τ_0_, and the effective energy barrier (*U*_eff_) correspond to the thermally assisted Orbach relaxation process, *C* and *n* are the parameters of the Raman relaxation process, while τ_QTM_ represents the relaxation time through the quantum tunneling of the magnetization (QTM).^48^ As shown in Figure 5B, the nonlinear Arrhenius plot signifies the contribution of Raman and QTM mechanisms at lower temperatures. At intermediate temperatures, the curvature of lnτ vs 1/*T* suggests dominance by the Raman process, while at even lower temperatures (<10 K) the relaxation time starts becoming temperature-independent. At high temperatures, the relaxation is governed by the thermally activated Orbach process, exhibiting exponential temperature dependence. Therefore, the best-fit to the experimental data in the 10–64 K temperature region afforded the following parameters: *U*_eff_ = 963.2(1) K, τ_0_ = 4.0(1) × 10^–11^ s, *C* = 0.186(2) s^–1^ K^–*n*^, *n* = 1.54(2), and τ_QTM_ = 4.4(1) × 10^–3^ s. The values of the parameters τ_0_, *C*, and *n* are within the expected range for mononuclear Dy^III^-based SMMs, while the exponent *n* of the Raman process takes a significantly smaller value than the expected for a Kramers ion (*n* = 9).^25^ However, it has been reported that deviations in the value of *n* suggest the mixing of optical and acoustic phonons, most probably due to the presence of low-energy optical phonons that contribute to the Raman demagnetization.^49^ The *U*_eff_ value closely aligns with those observed in structurally similar Dy^III^ molecular compounds featuring pentadentate N-donor Schiff-base macrocycles (Table S1). The reported *U*_eff_ of 1085(45) K for the structurally similar complex [Dy­(L^N5^)­(Ph_3_SiO)_2_]­(BPh_4_) is very close to the value obtained for **1-Dy** (although unspecified the selected for fitting high temperature regime), emphasizing the minimal effect of the counterion on the magnetization dynamics. Furthermore, the experimental *U*_eff_ value of the *D*_5h_ complex **1-Dy** is similar to that of the *D*_6h_ complex [Dy­(L^N6^)­(Ph_3_SiO)_2_]­(PF_6_) featuring the 2,6-diacetylpyridine ‘head’ unit (*U*_eff_ = 989 K),^25m^ while significantly higher than the *U*_eff_ of 779 K for compound [Dy­(L_phen_^N6^)­(Ph_3_SiO)_2_]­(PF_6_) bearing the 1,10-phenanthroline-2,9-dicarbaldehyde ‘head’ unit.^25n^

**Figure 5:**
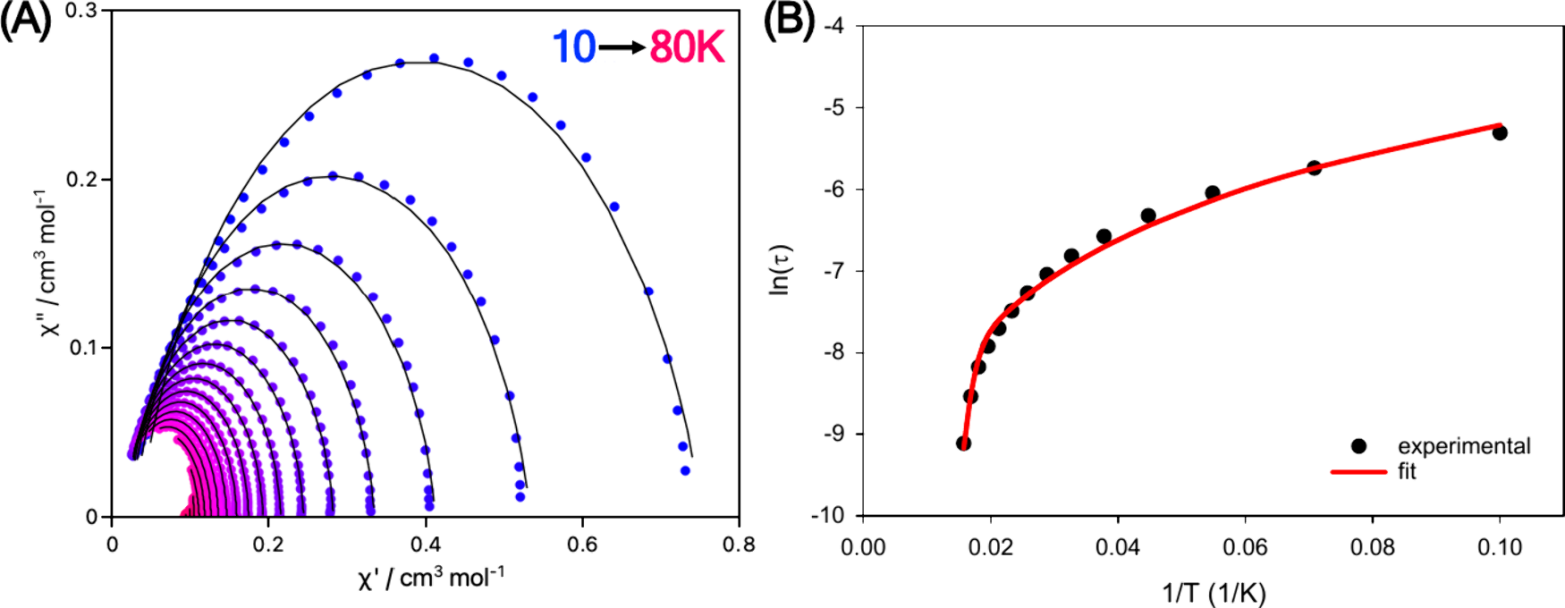
(A) Cole–Cole plots under zero applied dc field over the temperature range of 10–80 K for complex **1-Dy**. The solid lines are the best-fit of the data. (B) Temperature dependence of the relaxation times (τ) for **1-Dy**, where the red line represents the fit of the data through eq 1.

To further examine the distribution (α) of relaxation times across the entire temperature range, the Cole–Cole plots (Figure 5A) were fitted using a generalized Debye model (eqs S1 and S2, see ESI for details). The nonsemicircular plots suggest a broad distribution of relaxation times, likely due to the overlapping of different relaxation mechanisms. Indeed, high α values (0.18–0.14, Table S6) at the lowest temperatures (10–20 K) are supportive of such an overlap between Raman and QTM relaxation processes.

In contrast, ac magnetic susceptibility studies for the Ho analogue (**1-Ho**), carried out at the highest accessible frequency of 997 Hz, revealed no temperature-dependent out-of-phase signals, in the presence or absence of a dc field (Figures S11 and S12), clearly indicating QTM as the predominant relaxation mechanism. This is a common phenomenon in non-Kramers ions, and a fundamental difference compared to the Kramers analogues (i.e., **1-Dy**), where the symmetry of the ligand field can easily mix the wave functions of the *m*_*J*_ states leading to fast tunneling relaxation within the ground state, without observing any χ_Μ_″ peaks. Hence, the crystal field symmetry needs to be strictly designed in such systems. In the case of the oblate-shaped Ho^III^ ion, it has been predicted that the *U*_eff_ can be maximized with one or two linearly coordinated ligands, where favoring for ground-state QTM is insignificant due to the existence of negligible transverse anisotropies.^22a^ Such an approach has been successful only for the monodentate ligand design assembly, where five identical and soft groups (e.g., water, pyridine) occupy the *xy* plane, and two strong, closely packed ligands are located at the *z*-axis, thus creating mononuclear *D*_5h_ compounds with pseudolinear environment around the metal center.^20–22^

In complex **1-Ho** of this study, we have pioneered the use of a macrocycle chemical strategy to create a ‘soft’ equatorial plane via the chelate effect of a pentadentate Schiff-base ligand (L^N5^), while simultaneously reinforcing strong axiality with siloxide ligands. Although the high local symmetry of the Ho^III^, in accordance with the striking discrepancies in the axial Ho–O to Ho–N bond lengths in **1-Ho**, indicates a molecular system with enhanced axial anisotropy and large splitting of the *m*_*J*_ states, no signals of slow magnetization relaxation were observed at the ac measurements, even at the lowest operating temperature of 2 K. Previously reported high-performance mononuclear Ho^III^ SMMs display high *U*_eff_ values ranging from 290 to 715 K, with the compounds exhibiting nearly ideal *D*_5h_ molecular symmetry owing to the uniformity imposed by the monodentate equatorial ligands (Table S7). In the case of **1-Ho**, the macrocyclic ligand provides a slightly distorted pentagonal plane, while the different nature of N-donor atoms (pyridine, imine, and secondary amine) leads to an asymmetry of the electronic distribution around the oblate Ho^III^ ion. As a result, the pseudo-*D*_5h_ local symmetry of the metal ion does not match the overall molecular *C*_1_ point group symmetry of the coordination compound. Thereby, the transverse anisotropy, resulting from the crystal field effects, seems to efficiently enhance the tunneling relaxation. Furthermore, 5% dilution of the Ho^III^ complex in a diamagnetic Y^III^ matrix (**1-Ho@Y**) has led to an identical magnetic behavior (Figure S13), discarding any significant effects from intermolecular magnetic dipolar interactions on the ground state quantum tunneling of magnetization. The packing diagram of neighboring molecules in the crystal structure of **1-Ho@Y** revealed that the shortest intermetallic distance is 11.177(2) Å (Figure S14).

### 3.4 Single-Crystal Magnetic Hysteresis Studies

To further analyze the magnetization dynamics of **1-Dy**, and better understand the diversity in the mechanism of relaxation in **1-Ho**, magnetization (*M*) versus applied dc field (*H*) hysteresis studies were performed on single crystals of both complexes at temperatures down to 0.03 K, between ±1 T, with field sweep rates ranging from 0.128 to 0.001 T/s using a μ-SQUID apparatus. The time resolution was approximately 1 ms and the magnetic field was applied in different directions of the μ-SQUID plane with a precision better than 0.1° by driving three orthogonal coils separately.

In particular, single crystals of **1-Dy** and **1-Ho** were employed for the μ-SQUID studies, with the field-aligned along the easy axis of the crystal employing a 3D vector magnet and the transverse field method.^50^ Open temperature- and sweep-dependent loops were observed for **1-Dy**, with the typical butterfly shape obtained up to a temperature of 4 K at a sweep rate of 16 mT/s (Figure 6A,B). The sharp drop of magnetization that occurs at zero field, combined with a sharp peak in the first-field derivative (δ*M*/δ*H*) (Figure 6A,D), is due to ground state tunneling relaxation, leading to very small coercivities, which is a common phenomenon for mononuclear Dy^III^ SMMs.^51^ The blocking temperature extracted from the ZFC/FC magnetization plots is in perfect match with the experimentally observed temperature (4 K) from the μ-SQUID hysteresis studies.

**Figure 6:**
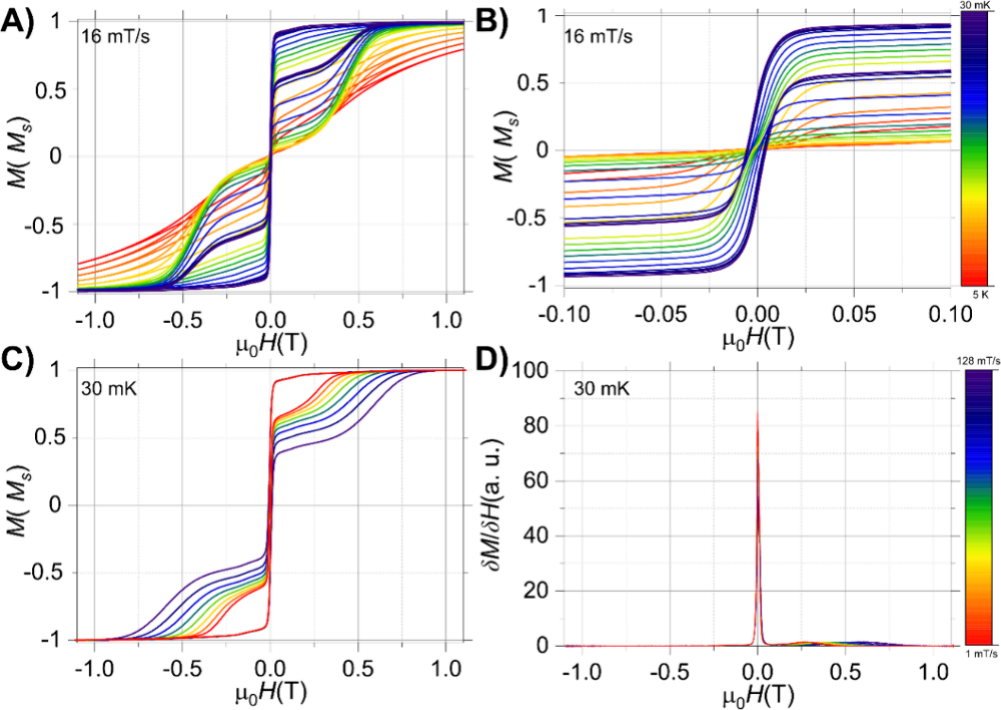
μ-SQUID hysteresis loops collected in a single crystal of **1-Dy** with the applied field along the easy axis of the crystal. (A) Loops collected with a sweep rate of 16 mT/s from 0.03 K up to 5 K; (B) magnification of the loops collected at 16 mT/s; (C) sweep-dependent hysteresis study conducted at 30 mK from 1 up to 128 mT/s; (D) derivative of the loops shown in panel (C).

Similarly, hysteresis studies were carried out for a single crystal of **1-Ho** with the field applied along the easy axis (Figure 7A). In contrast to **1-Dy**, the obtained loops in **1-Ho** have a rectangular shape between ±0.15 T (Figure 7D) with no drop of magnetization at zero field, resulting in significantly larger coercivity than **1-Dy**. In addition, compound **1-Ho** revealed a smaller sweep-rate dependence (Figure 7C), as well as a smaller temperature-dependence (Figure 7B), than that of **1-Dy** (Figure 6B,C). Moreover, the temperature-dependent study showed that the loops are open up to a temperature of 0.2 K, corroborating a smaller axial anisotropy in this system. Hence, the magnetization of **1-Ho** is completely blocked only below 0.2 K.

**Figure 7:**
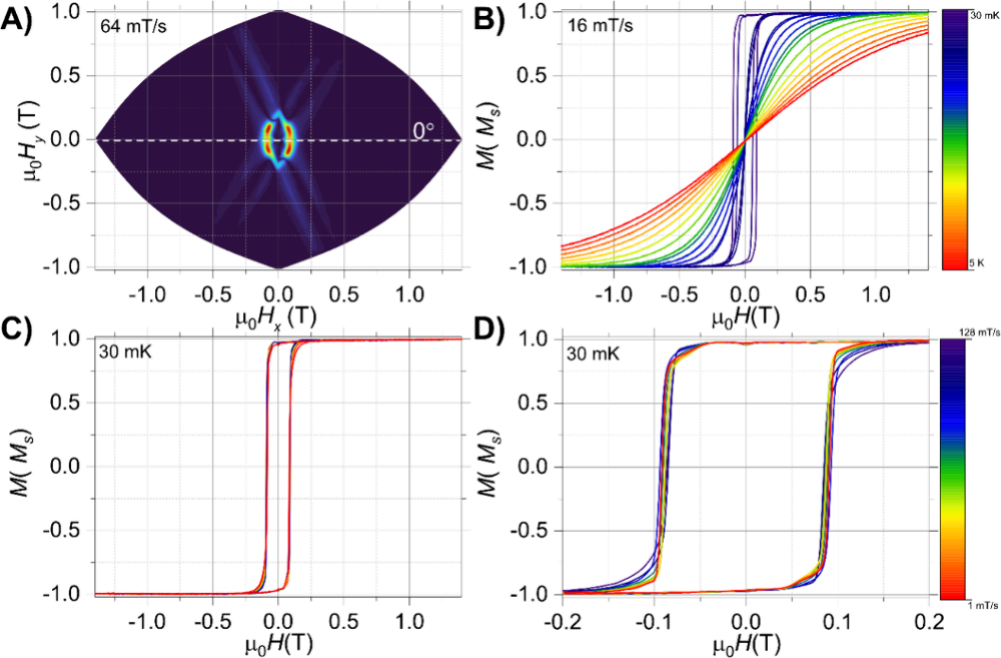
μ-SQUID hysteresis loops collected in a single crystal of **1-Ho** with the applied field along the easy axis of the crystal. (A) Angular map highlighting slightly different oriented molecules within the unit cell. The loops were collected with a sweep rate of 64 mT/s at 30 mK; (B) temperature-dependent loops with a sweep rate of 16 mT/s from 0.03 K up to 5 K; (C) field sweep study at 30 mK and sweep rates from 128 down to 1 mT/s; and (D) zoom of loops shown in panel (C).

The suppression of tunneling at zero-field has been observed in similar molecular systems, where the strong hyperfine interaction between the nuclear and electronic spin in Ho^III^ compounds, is the origin of shifting the zero-field step to the in-field area.^20^ Ho^III^ has a nuclear spin, *I* = 7/2, with almost 100% abundance, hence, this system is an excellent test subject to investigate the hyperfine-driven (hf-driven) QTM, which might be responsible for the quenched zero-field QTM observed in this system. To this end, a 5% diluted sample of Ho^III^ into the Y^III^ analogue (**1-Ho@Y**) was prepared and investigated through single-crystal magnetic hysteresis studies. Open loops were clearly visible for the **1-Ho@Y** sample until 0.2 K, with a staircase-like structure, characteristic of the hyperfine-driven QTM at certain fields (Figure 8D). However, the S-shaped loops observed in the case of **1-Ho@Y** are distinctly different than the rectangular ones noticed for the pure Ho^III^ compound (**1-Ho**). This can be attributed to the presence of differently oriented molecules in the unit cell and packing effects, as shown from the angular maps in Figures 7A and 8A. Indeed, neighboring molecules of **1-Ho** are packed in such a way that their easy magnetization axes, aligned with the O_ax_–Ho–O_ax_ molecular *z*-axis, form an angle of 36.8°, while the same angle in the **1-Y** analogue is 88.5° (Figure S15). This is most likely the origin of angular dependence of the applied magnetic field relative to the molecular easy axes, as observed in the hysteresis studies.

**Figure 8:**
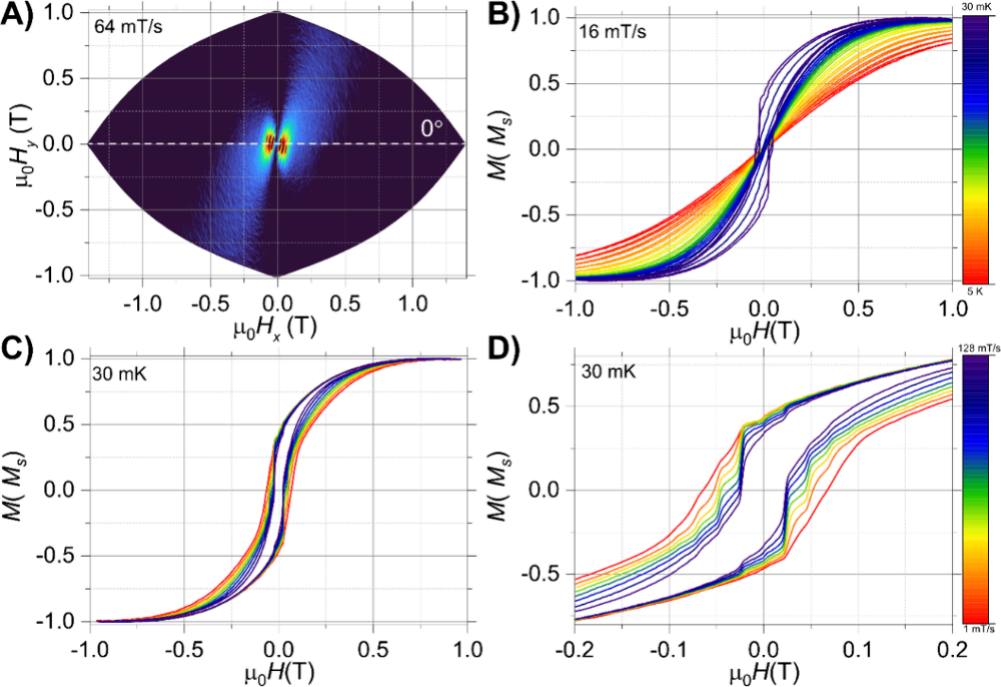
μ-SQUID hysteresis loops collected in a single crystal of **1-Ho@Y** with the applied field along the easy axis of the crystal. (A) Angular map highlighting a single molecule within the unit cell. The loops were collected with a sweep rate of 64 mT/s at 30 mK; (B) temperature-dependent loops with a sweep rate of 16 mT/s from 30 mK up to 5 K; (C) field sweep study at 30 mK and sweep rates from 128 down to 1 mT/s; and (D) zoom of loops shown in panel (C) highlight the hf-QTM processes.

From a theoretical perspective, for an ^165^Ho system the integer electronic spin *J* = 8, coupled with the half-integer nuclear spin *I* = 7/2, results in a splitting of the ground ±*m*_*J*_ state into 2 × (2*I* + 1) = 16 hyperfine ±*m*_*I*_ states with particular projections {±7/2, ±5/2, ±3/2, ±1/2}.^52^ Furthermore, the well-defined peaks in the derivative (δ*M*/δ*H*) of the hysteresis loop measured at 0.03 K with various field-sweep rates clearly indicate the presence of hyperfine-driven QTM steps (Figure 9C). The experimental crossings observed in the *M*(*H*) loops and δ*M*/δ*H* of **1-Ho@Y** can be mapped onto the Zeeman diagram of the holmium complex with a hyperfine interaction (*A*_hf_) of 0.028 cm^–1^ and the crystal field parameters obtained from CASSCF calculations $\left(\underset{k=2,4,6}{\sum }\overset{k}{\underset{q=0}{\sum }}B_{k}^{q}{Ô}_{k}^{q}\right)$ (see Table S12 for details). Comparison of the nuclear spin crossings in the Zeeman diagram and the experimental hf-QTM transitions show that the hyperfine crossings can be associated with QTM between the *m*_*J*_ = ±8 electronic state at Δ*m*_*I*_ = 0 and/or Δ*m*_*I*_ = ±1 nuclear spin-flip transitions (Figure 9B). Although Δ*m*_*I*_ = 0 are supposedly to be the only active ones, distortion in the crystal lattice has shown to induce Δ*m*_*I*_ ≠ 0 transitions as well.^52,53^

**Figure 9:**
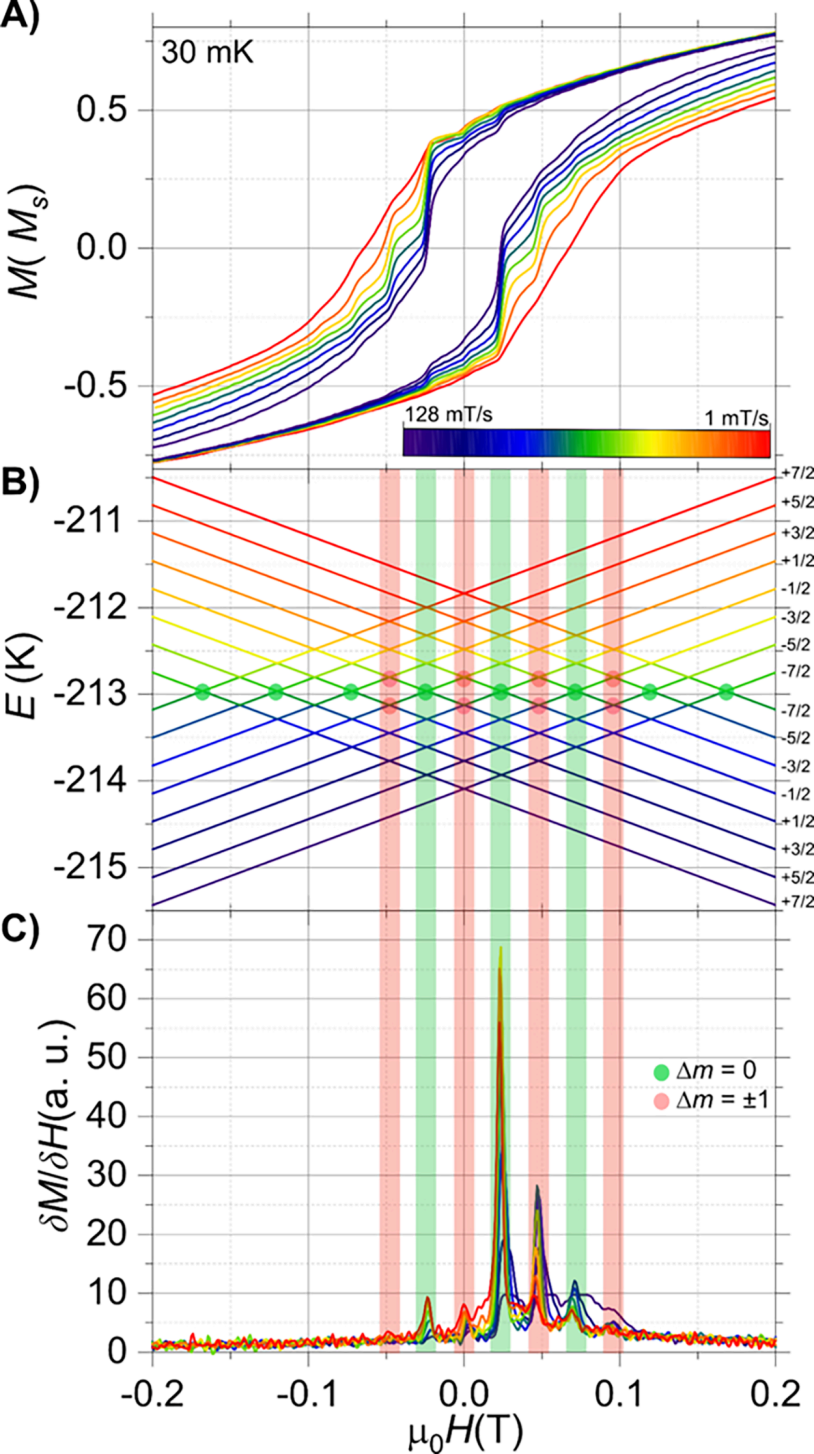
μ-SQUID hysteresis loops collected in a single crystal of **1-Ho@Y** with the applied field along the easy axis of the crystal. (A) Loops collected at 30 mK and different field-sweep rates; (B) Zeeman diagram for the holmium complex employing the crystal field parameters obtained from CASSCF and a hyperfine interaction, *A*_hf_, of 0.028 cm^–1^. (C) Derivative (δ*M*/δ*H*) of data shown in (A), highlighting the hyperfine-driven QTM processes.

### 3.5 Ab Initio Studies

For a precise evaluation of the magnetic characteristics and the lowest lying energy states of compounds **1-Dy** and **1-Ho**, ab initio calculations employing the CASSCF/SO-RASSI/SINGLE_ANISO approach implemented in the OpenMolcas package were carried out. As anticipated from the strong axial crystal field provided by the triphenylsiloxide ligands, the Dy^III^ ion in **1-Dy** exhibits a quite large overall energy splitting of 1281 K (Table S8). Moreover, the direction of the principal anisotropy axis of the *g*-tensor in the ground Kramer Doublet (KD) lies nearly parallel to the O···Dy···O axis, as depicted in Figure 10A. The calculated energies of the eight lowest KDs of the ^6^H_15/2_ ground multiplet of the Dy^III^ center along with the principal components of the *g*-tensors and the wavefunction compositions of the ground and excited KDs of **1-Dy** are tabulated in Table S8. Analysis of the calculated *g*-tensors and relative wave function compositions reveal a pure (∼100%) and highly anisotropic ground state (*m*_*J*_ = ±15/2) characterized by pronounced axiality (*g*_*z*_ = 17.8963, *g*_*y*_ = 0.0007, *g*_*x*_ = 0.0003). The first excited state (*m*_*J*_ = ±13/2) is located at ∼573 K above the ground state with a percentage composition of 97.8% and is still axial (*g*_*z*_ = 16.9432, *g*_*y*_ = 0.1080, *g*_*x*_ = 0.0963). The six following excited ±*m*_*J*_ states (KDs(3–8)) are highly mixed and close in energy, ranging from 905 to 1282 K; hence, the magnetic relaxation most likely proceeds from one of these excited states. In particular, KD3 lies at ∼ 905 K, and is characterized by an admixture of *m*_*J*_ = ±11/2 (17.8%), *m*_*J*_ = ±3/2 (18.6%) and *m*_*J*_ = ±1/2 (58.8%), while the axial *g*_*z*_ component still dominates over the transverse ones (*g*_*y*_, *g*_*x*_). The situation is reversed when transitioning to KD4 (∼993 K) and KD5 (∼1042 K), where the large values of the transverse *g*_*y*_ and *g*_*x*_ components dictate the nature of the doublets. Using the average matrix elements of the magnetic moment between the electronic states as a proxy for transition propensity, we predict that the greater probability transitioning from the KD2 → KD4 (Figure 11 and Table S9) leads to magnetic relaxation through the third excited state (KD4), which is very close to the experimentally determined *U*_eff_ value of 963 K.

**Figure 10:**
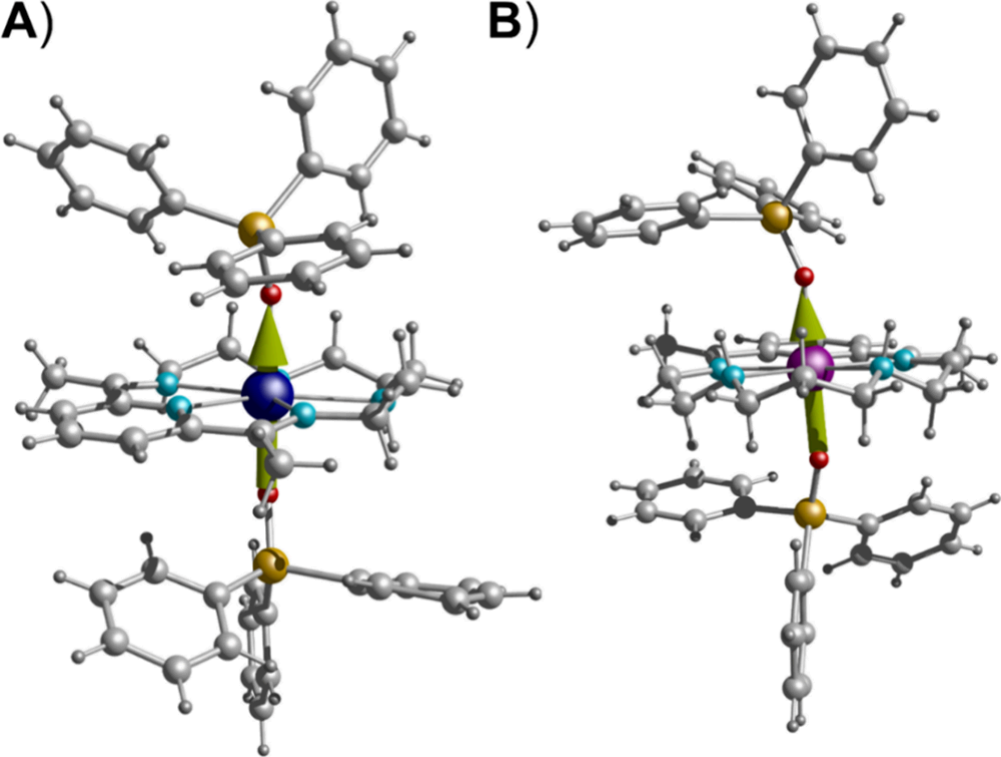
Direction of the principal magnetic anisotropy axis of the *g*-tensor in the ground Kramer doublet (green arrows) for compounds **1-Dy** (A) and **1-Ho** (B). Color code: Dy, dark blue; Ho, purple; O, red; C, light gray; N, pale blue; Si, orange; H, dark gray.

**Figure 11:**
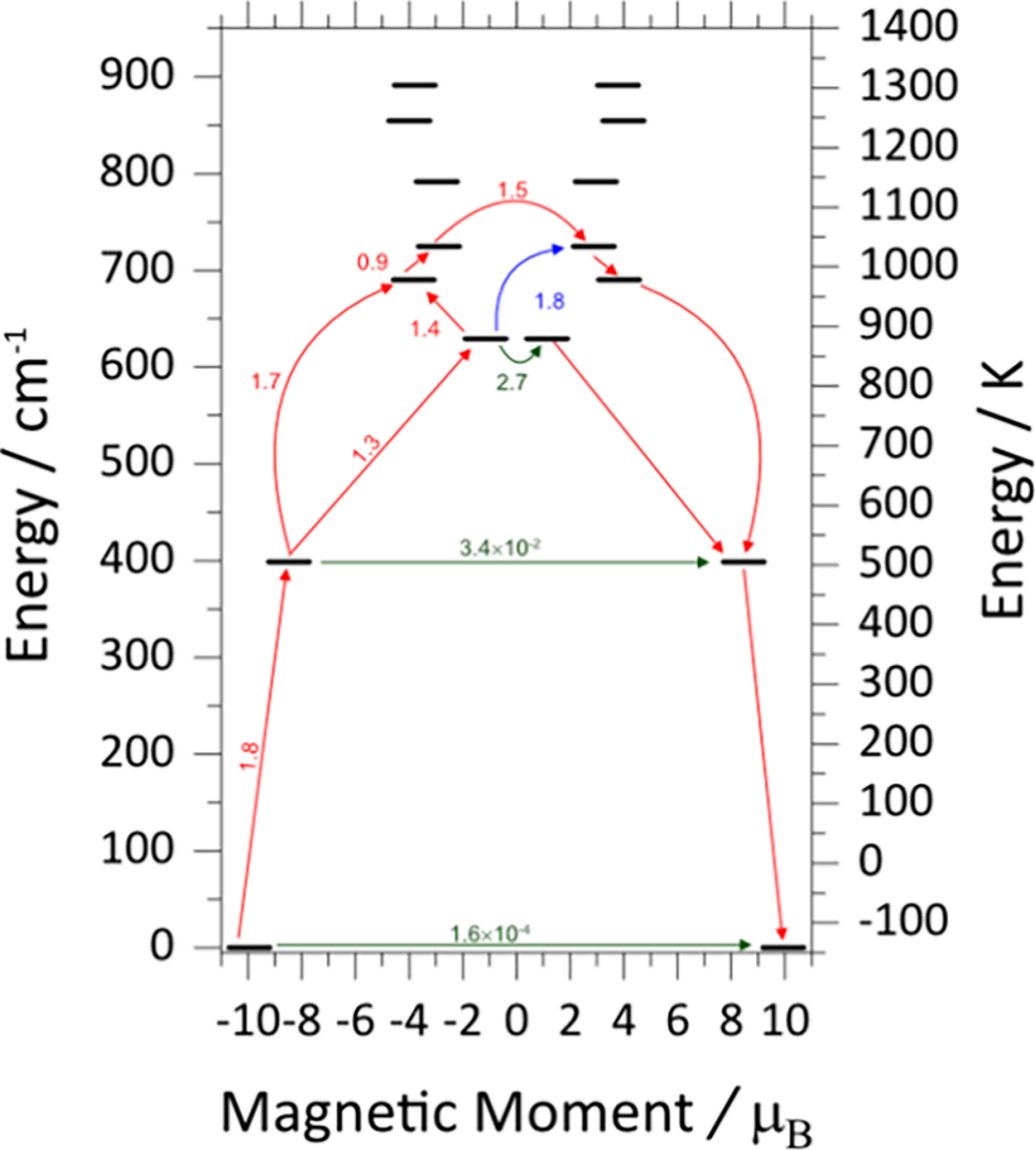
Energy level diagram of the low-lying doublets for **1-Dy**. The states are placed on the diagram according to their magnetic moments (bold black lines). The horizontal green lines show the tunneling transitions within each doublet state, while the nonhorizontal red and blue lines show the spin-phonon transition paths.

In the case of **1-Ho**, the wave function composition of the ground state is almost purely *m*_*J*_ = ±8 (99.6%) of a clear Ising-type (*g*_*z*_ = 19.8497) (Table S10). The main magnetic axis of the ground doublet is oriented along the O···Ho···O projection (Figure 10B), confirming the strong electrostatic potential generated by the axial deprotonated triphenysilanol ligands. Regardless of a large tunnel splitting (Δ_tun_) of ∼0.15 cm^–1^ in the ground state, due to the non-Kramers nature of Ho^III^, the ground state is not a pure doublet state, but rather a quasi-doublet (Table S10). Furthermore, the first excited quasi-doublet lies ∼ 429 K above the ground state with an appreciably large Δ_tun_ value of ∼5.2 cm^–1^ (Table S10), and a major contribution from the *m*_*J*_ = ±3 (71.6%) and a minor contribution from *m*_*J*_ = ±7 (11.4%) (highlighted with red color in Table S11). The following excited states are highly mixed and are distributed across a narrow range, spanning energy values from 456 to 623 K. These states exhibit tunneling gaps varying from 4.6 to 16.4 cm^–1^, classifying them as quasi-doublets (Table S10 and Table S11). It is worth noting that the calculated energy gap between the ground and first excited state in complex **1-Ho** is the second highest among all reported Ho^III^ SMMs with a *D*_5h_ symmetry (Table S7). This observation underscores the robust axial ligand field generated by the triphenylsiloxide ligands, concerted in a synergistic manner by the soft equatorial coordination induced by the L^N5^ macrocycle.

Although the ground quasi-doublet is well isolated from the remaining energy levels, no slow relaxation of magnetization was observed through the ac measurements, indicating a fast tunneling between the *m*_*J*_ = ±8 levels, without transcending to the first excited state. The striking difference between the previously reported mononuclear Ho^III^ SMMs and complex **1-Ho** is the large Δ_tun_ value within the ground state (Table S7). Universally, Landau, Zener, and Stuckelberg (LZS) first discussed the nonadiabatic transition between two states, and the tunneling probability for an avoided energy level crossing.^54^ The avoided crossing is related to the transverse anisotropy crystal field terms and transverse magnetic fields. At the crossing, the Hamiltonian’s eigenvectors form a linear combination of the positive and negative spin projections. As a result, there is a finite probability for the spin to exist on both sides of the barrier, enabling tunneling between states. According to the LZS model, the tunneling probability is directly proportional to the magnitude of the tunnel splitting. For non-Kramers ions, such as Ho^III^, the transverse crystal field plays a key role in generating a tunnel splitting.^55^

Murrie and co-workers theoretically predicted that in a similar pentadentate N-donor macrocycle (L_1_^N5^, Table S1), the negative charges at the secondary amine (>NH−) groups are nearly four times larger than those at the imine and pyridine N atoms, comparable to that found for the axial O atoms.^24b^ Consequently, the charges at the equatorial pentagonal plane of **1-Ho** are unevenly distributed, unlike previously reported *D*_5h_ Ho^III^ SMMs with identical equatorial ligands. This uneven distribution likely generates a large tunnel splitting in **1-Ho** through transverse crystal field interactions with the >NH- groups. Hence, despite the strong axial crystal field which stabilizes the *m*_*J*_ = ±8 ground state and creates a large separation between the ground and the excited states, the sizable tunneling gap within the ground doublet enables rapid quantum tunneling relaxation.

Inspection of the calculated crystal field parameters (*B*_*k*_^*q*^) revealed that the axial parameters (*B*_*k*_^0^, *k* = 2, 4, 6) dominate over the transverse ones (*B*_*k*_^*q*^, *q* ≠ 0 and *k* = 2, 4, 6) for both compounds, as shown in Table S12. This is expected due to the strong ligation by the hard O-donor atoms of the triphenylsilanolates along the easy axis. In particular, the magnitude of |*B*_2_^0^| dictates the molecular compound’s axiality and the crystal field splitting scale. Thereby, the |*B*_2_^0^| value for **1-Dy** is three times higher than that of **1-Ho**, reflecting the larger energy separation of the crystal field-induced states in **1-Dy**.

Finally, besides the fact that **1-Ho** does not exhibit SMM behavior at high temperatures, its intrinsic characteristics as a molecular system resulting from the crystal field environment and the nature of the Ln^III^, can be further exploited as a potential spin qubit. According to Coronado and co-workers, two minimal electronic prerequisites for having a spin qubit are the controlled mixing of the wavefunctions in a well-defined level subset and the sufficient isolation of this subset from the remaining energy spectrum.^56^ For non-Kramers Ln^III^ ions, a large tunneling gap (0.1–1 cm^–1^) is essential to align with working frequencies of the microwave technologies (1–100 GHz), for the easier manipulation of the quantum state and for diminishing decoherence.^6c^ Therefore, to our sense, complex **1-Ho** fulfills the above criteria and can be further examined as a prospective lanthanide-based spin qubit. These results will be reported in due course.

## 4 Conclusions

In summary, we have reported a detailed comparative magnetic study of the mononuclear lanthanide macrocyclic complexes with the general formulas [Ln­(L^N5^)­(Ph_3_SiO)_2_]­(PF_6_), where Ln^III^ = Dy^III^ (**1-Dy**) and Ho^III^ (**1-Ho**), exploiting experimental evidence from bulk and single-crystal hysteresis studies, as well as supportive results from ab initio calculations. The in situ prepared [1+1] Schiff-base macrocyclic ligand, L^N5^, has formed a pentagonal N-rich equatorial plane around the metal centers, while the apical sites were occupied by two strongly bound, O-donor triphenylsiloxide ligands, leading to pseudo-*D*_5h_ local metal symmetry. Interestingly, complex **1-Dy** demonstrates slow relaxation of the magnetization below 64 K and a high *U*_eff_ value of 963 K at zero applied dc field, while the Ho^III^ congener (**1-Ho**) exhibits no out-of-phase signals at *T* > 2 K. Single-crystal hysteresis studies revealed that the magnetization blocking for **1-Dy** and **1-Ho** emerges below 4 and 0.2 K, respectively. Dilution of the Ho^III^ complex in a diamagnetic Y^III^ matrix (**1-Ho@Y**) revealed a step-like behavior in hysteresis loops driven by the hyperfine interactions at the field of avoided level crossing where the resonant tunneling occurs. Additionally, ab initio calculations were performed to unravel the low-lying crystal field-induced energy states of both compounds. The ground ±*m*_*J*_ states with the highest value were stabilized for both compounds due to the strong axial crystal field interaction, which also contributes to the large energy separation between the ground and the excited sublevels. Surprisingly, compound **1-Ho** exhibits a huge energy gap of ∼429 K, but the presence of a large tunneling splitting (Δ_tun_) of 0.15 cm^–1^ eventually leads to fast tunneling relaxation within the ground state, and as a result, the magnetization is trapped in the lowest *m*_*J*_ = ±8 quasi-doublet.

The experimental observations from this study underscore the critical role of molecular symmetry over local metal symmetry in determining the magnetization relaxation behavior of lanthanide complexes incorporating Kramers (Dy^III^) and non-Kramers (Ho^III^) ions when employing the [1+1] Schiff-base macrocycle approach. The asymmetrical distribution of the N atoms’ local charges at the equatorial plane, introduced by the macrocycle L^N5^, appears to have minimal impact on the SMM dynamics of the Dy^III^ complex. However, shifting to the Ho^III^ isostructural analogue, this asymmetry appears to significantly affect the thermally assisted relaxation processes, as quantified by the appreciably smaller |*B*_2_^0^| crystal-field term, thus facilitating the QTM mechanism for the spin reversal. We are currently seeking ways to further characterize **1-Ho** (and **1-Ho@Y**) by employing more advanced techniques, such as time-resolved spectroscopies and pulsed Electron Paramagnetic Resonance (EPR), and elucidate the viability of this molecular system as a potential spin qubit.

## Supplementary Material



## Notes

Crystallographic data, bond distances and angles, IR and p-XRD spectra, structural and supramolecular figures, magnetism (bulk and single-crystal) plots, and data from the theoretical calculations performed on complexes **1-Dy** and **1-Ho** (PDF)
