# Supplementary material for: Deciphering the Magnetostructural Criteria in DyIII and HoIII Macrocycle-Based Single-Molecule Magnets with Pseudo‑D 5h Symmetry: A Combined Single-Crystal Hysteresis and Theoretical Study
Source: Inorg Chem. 2025 Jun 5;64(23):11476–89. doi: 10.1021/acs.inorgchem.5c00644 (PMC12175138; doi:10.1021/acs.inorgchem.5c00644)
Supplement: Supplementary file 1 [file ic5c00644_si_001.pdf]

Supporting Information

Deciphering the Magnetostructural Criteria in Dy^{III} and Ho^{III} Macrocycle-Based Single-Molecule Magnets with Pseudo-D_{5h} Symmetry: A Combined Single-Crystal Hysteresis and Theoretical Study

Alexandros S. Armenis,[a] Georgia P. Bakali,[a] Sagar Paul,[b] Konstantinos N. Pantelis,[a] Luís Cunha-Silva,[c] Jinkui Tang,[d] Dimitris I. Alexandropoulos,[a] Wolfgang Wernsdorfer,[b,e] Eufemio Moreno-Pineda,*[,b,f,g] Theocharis C. Stamatatos*[,a,h]

[a] Department of Chemistry, University of Patras, 265 04 Patras, Greece

[b] Physikalisches Institut, Karlsruhe Institute of Technology (KIT), Kaiserstraße 12, Karlsruhe, D-76131, Germany

[c] LAQV/REQUIMTE & Department of Chemistry and Biochemistry, Faculty of Sciences, University of Porto, 4169-007 Porto, Portugal

[d] State Key Laboratory of Rare Earth Resource Utilization, Changchun Institute of Applied Chemistry, Chinese Academy of Sciences, Changchun 130022, P. R. China

[e] Institute for Quantum Materials and Technology (IQMT), Karlsruhe Institute of Technology (KIT), Hermann-von-Helmholtz-Platz 1, Eggenstein-Leopoldshafen, D-76344, Germany

[f] Universidad de Panamá, Facultad de Ciencias Naturales, Exactas y Tecnología, Depto. de Química-Física, 0824 Panamá, Panamá.

[g] Universidad de Panamá, Facultad de Ciencias Naturales, Exactas y Tecnología, Grupo de Investigación de Materiales, 0824 Panamá, Panamá.

[h] Institute of Chemical Engineering Sciences, Foundation for Research and Technology – Hellas (FORTH/ICE – HT), Platani, P.O. Box 1414, 26504 Patras, Greece

Corresponding authors: Prof. Theocharis C. Stamatatos – Prof. Eufemio Moreno-Pineda

E-mails: thstama@upatras.gr & eufemio.moreno@up.ac.pa

1. Structural and Spectroscopic Characterization

A. Infrared Spectra

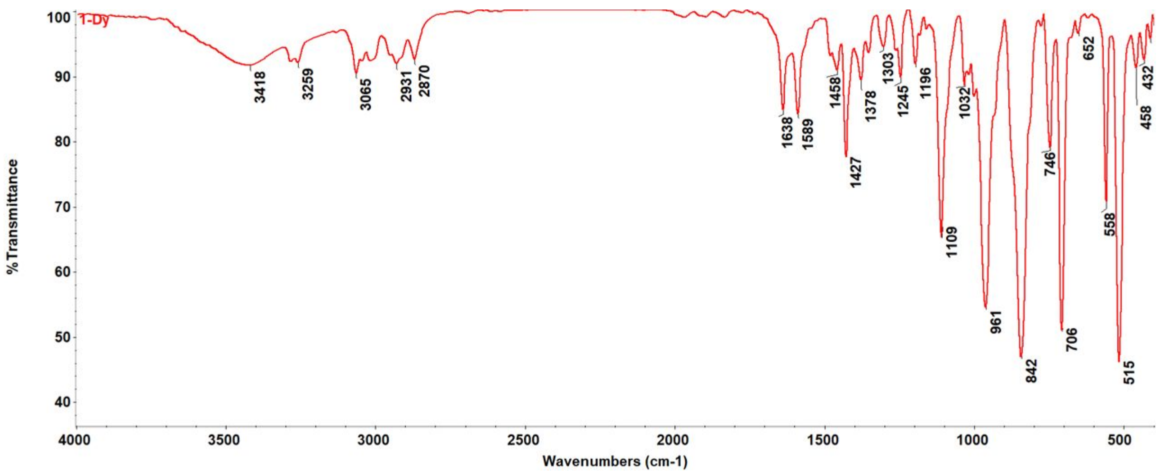

Figure S1. FT-IR spectrum of compound **1-Dy**.

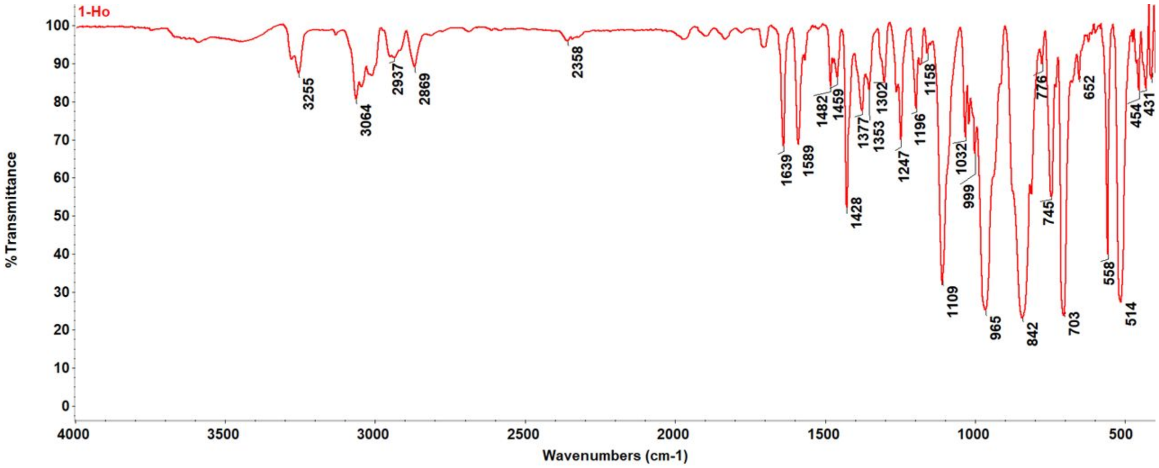

Figure S2. FT-IR spectrum of compound **1-Ho**.

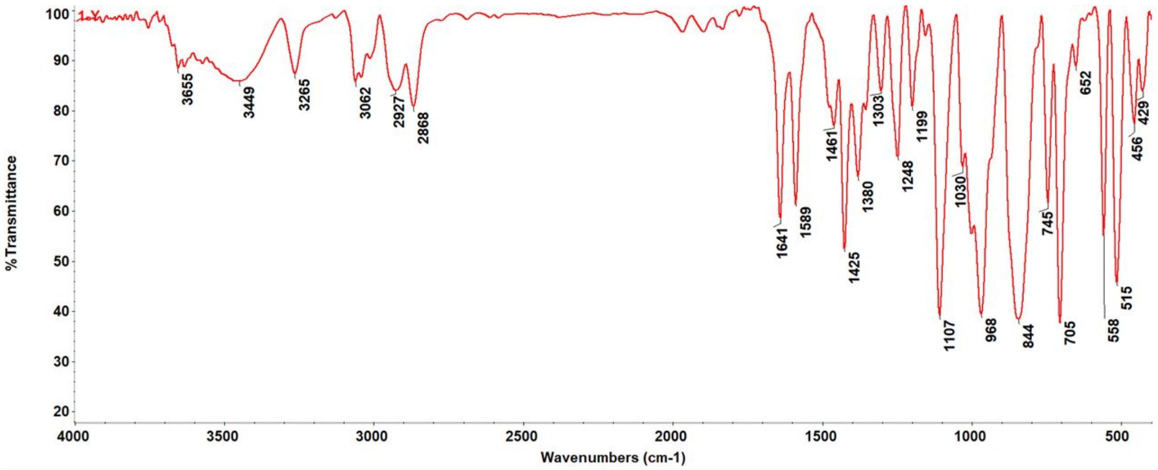

Figure S3. FT-IR spectrum of compound **1-Y**.

IR discussion: All reported compounds are structurally similar and, as a result, exhibit identical IR spectra (Figures S1-S3). In detail, the medium intensity bands at □1640 cm^{-1} and □1589 cm^{-1} are attributed to the stretching frequencies of C=N bonds of the formed imine bonds and the pyridyl subunit of the macrocyclic ligand, respectively.[S1,S2] Accordingly, the secondary amine (-NH-) groups contained in the Schiff-base macrocycle are reflected by the presence of a medium intensity band in the 3255-3265 cm^{-1} range.[S1,S2] Furthermore, the presence of the PF_6^- counterion is confirmed by the strong intensity band at □842 cm^{-1}, which is ascribed to the P-F bond stretching.[S3] Moreover, the coordination of the triphenylsiloxide ligands is verified by the strong bands in regions 930−1150 and 515-560 cm^{-1}, representing Si−O stretching and bending/rocking vibrational modes, respectively.[S4]

Table S1. All the previously reported mononuclear Dy^{III} complexes containing [1+1] Schiff-base macrocycles with pentagonal bipyramidal pseudo-symmetry.

**Compound**	**Macrocycle Structure**	$\boldsymbol{U_{eff}}$ **[K] / field [Oe]**	**CShM for** $\boldsymbol{D_{5h}}$	**Ref.**
$[Dy(L_1^{N5})(Ph_3SiO)_2](BPh_4)$	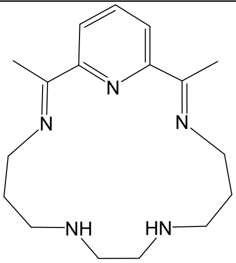	1108 / 0	Dy1: **1.293**<br>Dy2: **1.681**	24b
$[Dy(L_2^{N5})(\mathit{R/S}\text{-BINOL})_2](BPh_4)$	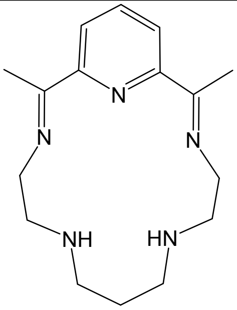	403(4) / 0	*R*-Dy: **1.680**<br>*S*-Dy: **1.690**	25d
$[Dy(L_2^{N5})(Ph_3SiO)_2](BPh_4)$	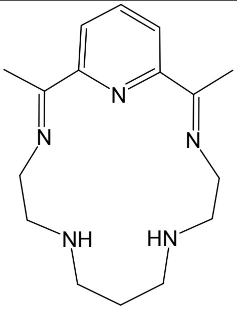	1085(45) / 0	Dy1: **1.241**<br>Dy2: **1.390**	25k
$[Dy(L_2^{N5})(Cl_2NO_2\text{-}PhO)_2)_2](BPh_4)$	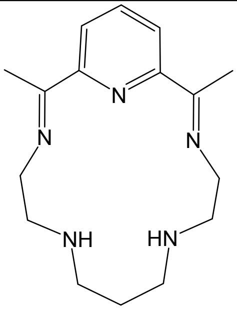	39(3) / 200	Dy1: **1.235**<br>Dy2: **1.071**	25k

$[Dy(L_2^{N5})(F_2\text{-}PhO)_2](BPh_4)$	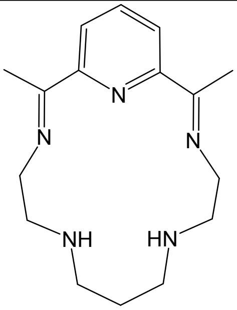	42(0.5)<br>5% Y-doped	Dy1: **1.207**	251
$[Dy(L_2^{N5})(Ph_3SiO)_2](PF_6)$	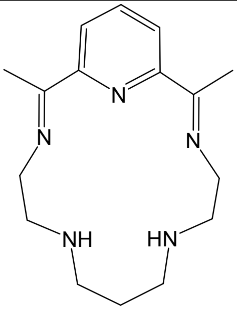	1038 / 0	Dy1: **1.085**	t.w.

Ligands' Abbreviations: L_1^{N5} = (2E,14E)-2,15-dimethyl-3,7,10,14-tetraaza-1(2,6)-pyridinacyclopentadecaphane-2,14-diene; L_2^{N5} = (2E,13E)-2,14-dimethyl-3,6,10,13-tetraaza-1(2,6)-pyridinacyclotetradecaphane-2,13-diene; Ph_3SiO^- = triphenylsilanolate; *R*/*S*-BINOL$^-$ = monodeprotonated *R*/*S*-1-(2-hydroxynaphthalen-2-ol); F_2-PhO$^-$ = 3,5-difluorophenolate; Cl_2NO_2-PhO$^-$ = 2,6-dichloro-4-nitrophenolate; t.w. = this work.

B. X-Ray Crystallographic details

Table S2. Crystallographic data for compounds **1-Dy**, **1-Ho**, and **1-Y**.

Parameter	**1-Dy**	**1-Ho**	**1-Y·CH₂Cl₂**
Formula	$C_{103}H_{109}Dy_2F_{12}N_{10}O_4P_2Si_4$	$C_{52}H_{55}HoF_6N_5O_2PSi_2$	$C_{53}H_{57}YF_6N_5O_2PSi_2Cl_2$
F_w / g·mol^{-1}	2278.30	1148.09	1156.99
Crystal system	Monoclinic	Monoclinic	Monoclinic
Space group	$P2_1/c$	$P2_1/c$	$C2/c$
a / Å	18.0302(11)	18.0216(3)	34.0983(3)
b / Å	14.2522(9)	14.2866(1)	11.1774(1)
c / Å	21.3578(12)	21.3895(3)	29.8174(3)
α / °	90.0	90.0	90.0
β / °	114.551(2)	114.517(2)	101.813(1)

γ / °	90.0	90.0	90.0
V / Å^3	4992.1(5)	5010.57(14)	11123.63(18)
Z	4	4	8
T / K	150(2)	180.0(1)	180.0(1)
Radiation / λ (Å)	Mo Kα / 0.71073	Cu Kα / 1.54184	Cu Kα / 1.54184
ρ_{calcd} / g cm^{-3}	1.516	1.522	1.382
Reflections collected/unique (R_{int})	101705/10000 (0.0337)	32661/10013 (0.0243)	42441/11254 (0.0342)
Reflections with I>2σ(I)	9099	9058	9562
No. of parameters	718	827	633
R_1 [I>2σ(I)], wR_2 [I>2σ(I)]^{a,b}	0.0380, 0.0954	0.0398, 0.1015	0.0635, 0.1786
R_1 (all data), wR_2 (all data)^{a,b}	0.0425, 0.1001	0.0437, 0.1039	0.0724, 0.1871
$(\Delta/\sigma)_{max}$	0.001	0.001	0.001
$\Delta\rho_{max}/\Delta\rho_{min}$ (e Å^{-3})	2.255/-1.391	0.703/-0.999	1.057/-0.804
CCDC number	2421383	2422074	2422075

^{a} $R_1 = \Sigma(|F_o| - |F_o|) / \Sigma(|F_o|)$; ^{b} $wR_2 = \{\Sigma[w(F_o^2 - F_c^2)^2] / \Sigma[w(F_o^2)^2]\}^{1/2}$, $w = 1 / [\sigma^2(F_o^2) + (aP)^2 + bP]$, where $P = [\max(F_o^2, 0) + 2 F_c^2] / 3$.

Table S3. Selected bond distances (Å) and angles (°) for complex **1-Dy**.

**Bond distances (Å)**	
Dy1-N1	2.431(4)
Dy1-N2	2.446(3)
Dy1-N3	2.467(4)
Dy1-N4	2.492(4)
Dy1-N5	2.461(3)
Dy1-O1	2.168(3)
Dy1-O2	2.167(2)
**Bond angles (°)**	
O1-Dy1-O2	175.6(1)
N1-Dy1-N2	65.9(1)

N2-Dy1-N3	71.3(1)
N3-Dy1-N4	87.0(1)
N4-Dy1-N5	70.1(1)
N5-Dy1-N1	65.8(1)
O1-Dy1-N1	93.9(1)
O1-Dy1-N2	88.5(1)
O1-Dy1-N3	88.1(1)
O1-Dy1-N4	90.7(1)
O1-Dy1-N5	93.1(1)
O2-Dy1-N1	90.2(1)
O2-Dy1-N2	91.9(1)
O2-Dy1-N3	87.9(1)
O2-Dy1-N4	87.3(1)
O2-Dy1-N5	89.9(1)

Table S4. Selected bond distances (Å) and angles (°) for complex **1-Ho**.

**Bond distances (Å)**	
Ho1-N1	2.418(2)
Ho1-N2	2.450(2)
Ho1-N3	2.478(2)
Ho1-N4	2.452(2)
Ho1-N5	2.435(2)
Ho1-O1	2.163(2)
Ho1-O2	2.162(2)
**Bond angles (°)**	
O1-Ho1-O2	174.8(8)
N1-Ho1-N2	66.0(8)
N2-Ho1-N3	69.9(9)
N3-Ho1-N4	86.7(1)
N4-Ho1-N5	71.2(9)

N5-Ho1-N1	66.0(8)
O1-Ho1-N1	94.4(8)
O1-Ho1-N2	93.3(8)
O1-Ho1-N3	90.4(9)
O1-Ho1-N4	87.8(9)
O1-Ho1-N5	88.7(8)
O2-Ho1-N1	90.5(8)
O2-Ho1-N2	90.0(8)
O2-Ho1-N3	87.0(9)
O2-Ho1-N4	87.4(9)
O2-Ho1-N5	91.9(8)

Table S5. Continuous Shape Measures (CShM) values for the potential coordination polyhedra of the 7-coordinate Ln^{III} centers in complexes **1-Dy** and **1-Ho**.

Polyhedron[a,b]	**1-Dy**	**1-Ho**
HP	33.19	33.26
HPY	20.87	20.80
PBPY	**1.08**	**1.06**
COC	7.17	7.10
CTPR	5.84	5.78
JPBPY	2.68	2.69
JETPY	21.81	21.71

[a]Abbreviations: HP, Heptagon; HPY, Hexagonal Pyramid; PBPY, Pentagonal Bipyramid; COC, Capped Octahedron; CTPR, Capped Trigonal Prism; JPBPY, Johnson Pentagonal Bipyramid; JETPY, Johnson elongated triangular pyramid. [b]The value in boldface indicates the closest polyhedron according to the Continuous Shape Measures.

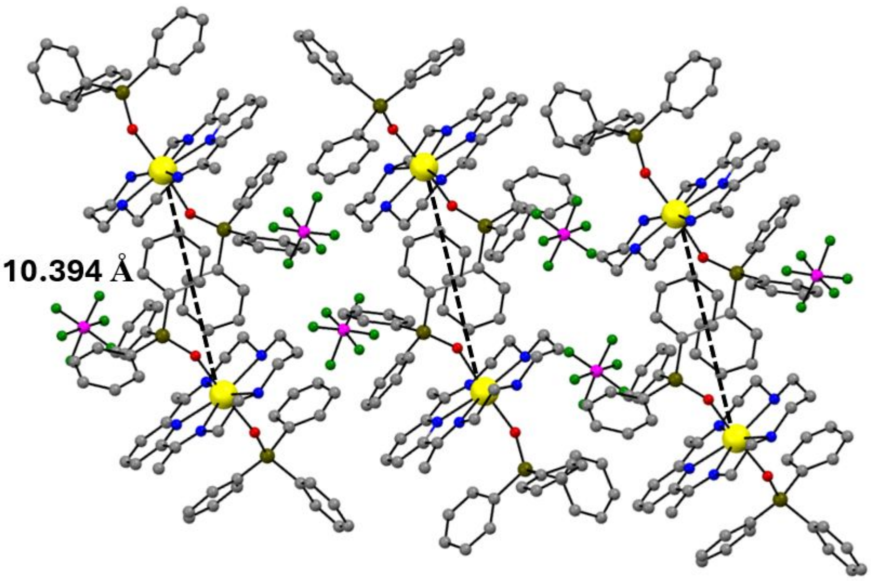

Figure S4. A portion of the repeating monomeric complexes in the crystal of **1-Dy** viewed along the *c*-axis and visualization of the shortest intermolecular Dy···Dy distance as black dashed lines. Color scheme: Dy^{III}, yellow; O, red; N, blue; C, grey; Si, olive; P, magenta; F, dark green. H-atoms are omitted for clarity.

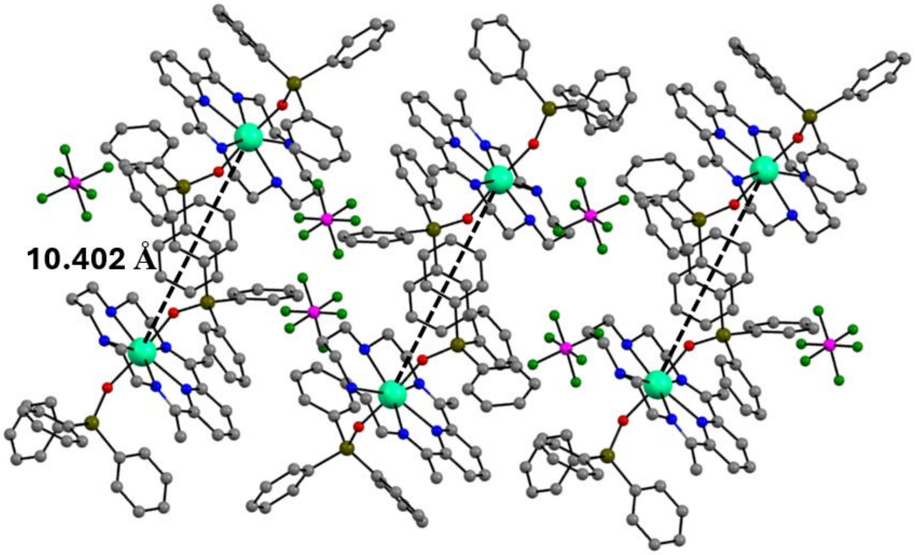

Figure S5. A portion of the repeating monomeric complexes in the crystal of **1-Ho** viewed along the *c*-axis and visualization of the shortest intermolecular Ho···Ho distance as black dashed lines. Color scheme: Ho^{III}, green; O, red; N, blue; C, grey; Si, olive; P, magenta; F, dark green. H-atoms are omitted for clarity.

2. Magnetic Measurements

Table S6. Relaxation fitting parameters for complex **1-Dy**.

T / K	χ_S / cm^3 mol^{-1}	χ_T / cm^3 mol^{-1}	τ / s	α	Residual
10.00	3.37E-02	7.53E-01	4.38E-03	1.80E-01	1.40E-03
14.12	2.75E-02	5.33E-01	3.21E-03	1.41E-01	9.38E-04
18.24	2.34E-02	4.12E-01	2.36E-03	1.14E-01	5.79E-04
22.35	2.07E-02	3.36E-01	1.79E-03	9.51E-02	3.41E-04
26.47	1.88E-02	2.83E-01	1.39E-03	8.10E-02	2.10E-04
30.59	1.77E-02	2.45E-01	1.10E-03	6.88E-02	1.23E-04
34.71	1.70E-02	2.16E-01	8.71E-04	5.83E-02	6.41E-05
38.83	1.60E-02	1.93E-01	6.95E-04	4.99E-02	3.97E-05
42.94	1.55E-02	1.75E-01	5.58E-04	4.32E-02	2.48E-05
47.06	1.53E-02	1.60E-01	4.50E-04	3.77E-02	1.44E-05
51.18	1.56E-02	1.47E-01	3.62E-04	3.25E-02	1.03E-05
55.30	1.58E-02	1.36E-01	2.80E-04	2.63E-02	8.57E-06
59.42	1.61E-02	1.27E-01	1.95E-04	2.37E-02	4.51E-06
63.53	1.51E-02	1.19E-01	1.10E-04	2.93E-02	5.99E-06
67.65	4.61E-12	1.12E-01	4.27E-05	4.87E-02	3.42E-06
71.77	9.76E-12	1.05E-01	1.89E-05	1.22E-02	1.11E-05
75.89	1.54E-11	9.98E-02	8.21E-06	3.84E-15	1.24E-05
80.01	2.75E-11	9.48E-02	3.70E-06	5.51E-15	1.04E-05

The reported parameters in **Table S6** were obtained by fitting the data through the following equations:

$$\chi'(v)=\chi_S+\frac{(\chi_T-\chi_S)[1+(2\pi v\tau)^{(1-\alpha)}\sin(\frac{\alpha\pi}{2})]}{1+2(2\pi v\tau)^{(1-\alpha)}\sin\left(\frac{\alpha\pi}{2}\right)+(2\pi v\tau)^{2(1-\alpha)}} \quad \textbf{(Equation S1)}$$

$$\chi''(v)=\frac{(\chi_T-\chi_S)(2\pi v\tau)^{(1-\alpha)}\cos(\frac{\alpha\pi}{2})}{1+2(2\pi v\tau)^{(1-\alpha)}\sin\left(\frac{\alpha\pi}{2}\right)+(2\pi v\tau)^{2(1-\alpha)}} \quad \textbf{(Equation S2)}$$

Table S7. Comparative study of all previously reported pentagonal bipyramidal Ho^{III} mononuclear complexes featuring SMM properties.

**Complex**	**CShM for D_{5h}**	**U_{eff} [K]**	**ΔE between ground and 1st excited state [K]**	**Ground state / Δ_{tun} [cm^{-1}]**	**Ref.**
$[Ho(CyPh_2PO)_2(H_2O)_5]I_3$	0.160	341(5.8)	286.3	$m_J = \pm 8$ / 0.0003	20
$[Ho(^tBuPO(NH^iPr)_2)_2(H_2O)_5]I_3$	0.20	355	374.7	$m_J = \pm 8$ / 0.001	22a
$[Ho(OSi(CH_3)_3)_2(py)_5](BPh_4)$	0.754	715(6)	502.1	$m_J = \pm 8$ / 0.0005	21
$[Ho(OCH(CH_3)C_6H_5)_2(py)_5](BPh_4)$	0.778	499(3)	356.2	$m_J = \pm 8$ / 0.002	21
$[Ho(OC_6H_3(CH_3)_2)_2(py)_5](BPh_4)$	0.881	397(12)	300.7	$m_J = \pm 8$ / 0.008	21
$[Ho(HMPA)_2(H_2O)_5]Cl_3$	0.154 (Ho1)<br>0.127 (Ho2)	290	-	-	22b
$[Ho(HMPA)_2(H_2O)_5]Br_3$	0.105	320	-	-	22b
$[Ho(tprpo)_2(H_2O)_5]Cl_3$	0.112	351	-	-	22c
$[Ho(tprpo)_2(H_2O)_5]Br_3$	0.132	418	-	-	22c
$[Ho(L^{N5})(Ph_3SiO)_2](PF_6)$	1.065	-	429.2	$m_J = \pm 8$ / 0.1485	t.w.

Abbreviations: $CyPh_2PO$ = dicyclohexyl(phenyl)phosphine oxide; $^tBuPO(NH^iPr)_2$ = organophosphonic diamide; $(CH_3)_3SiO^-$ = trimethylsilanolate; $(CH_3)C_6H_5CHO^-$ = 3,5-dimethylphenolate; $(CH_3)_2C_6H_3O^-$ = (R)-(+)-1-phenylethanolate; HMPA = hexamethylphosphoramide; tprpo: trispyrrolidinophosphineoxide; L^{N5} = (2E,13E)-2,14-dimethyl-3,6,10,13-tetraaza-1(2,6)-pyridinacyclotetradecaphane-2,13-diene; Ph_3SiO^- = triphenylsilanolate; t.w. = this work.

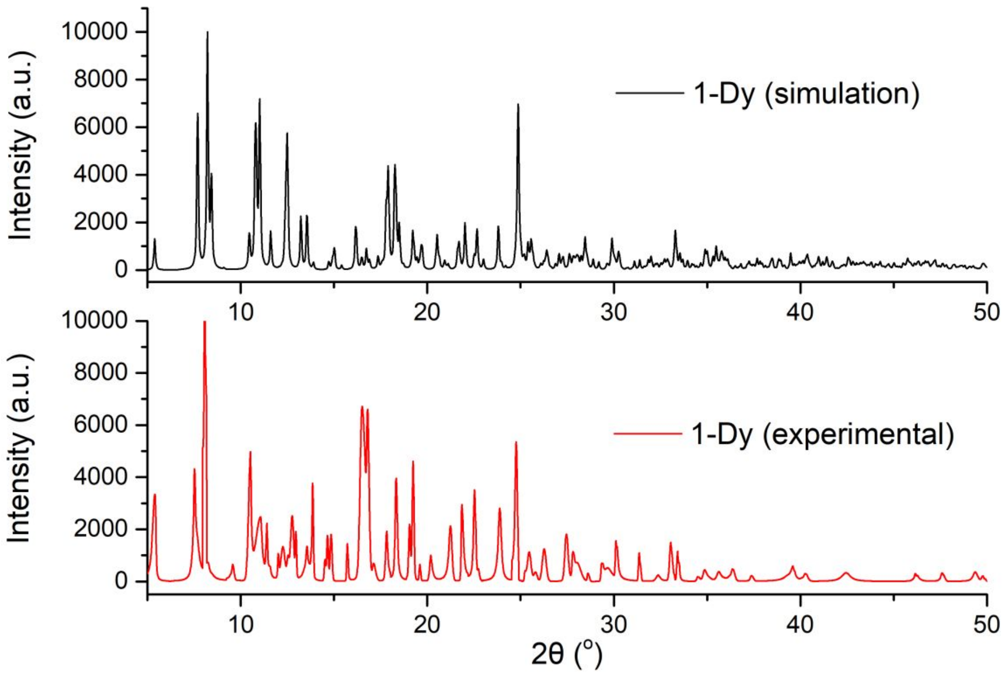

Figure S6. Experimental and simulated powder X-ray diffraction (p-XRD) patterns of **1-Dy**. The simulated pattern is calculated based on the structural model from the single-crystal X-ray diffraction data.

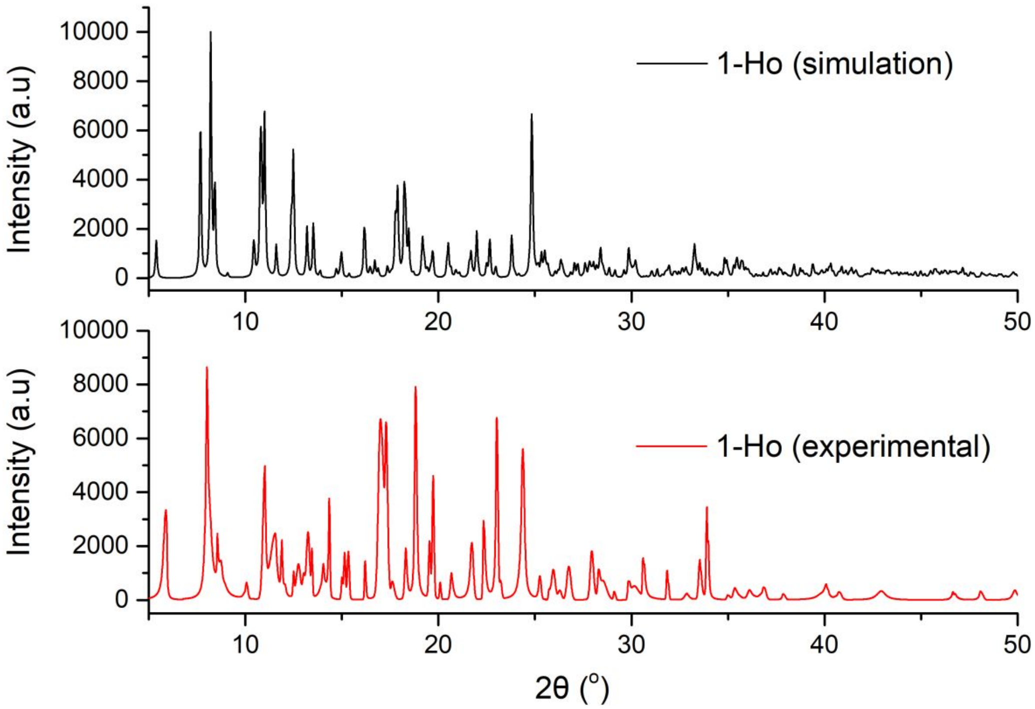

Figure S7. Experimental and simulated powder X-ray diffraction (p-XRD) patterns of **1-Ho**. The simulated pattern is calculated based on the structural model from the single-crystal X-ray diffraction data.

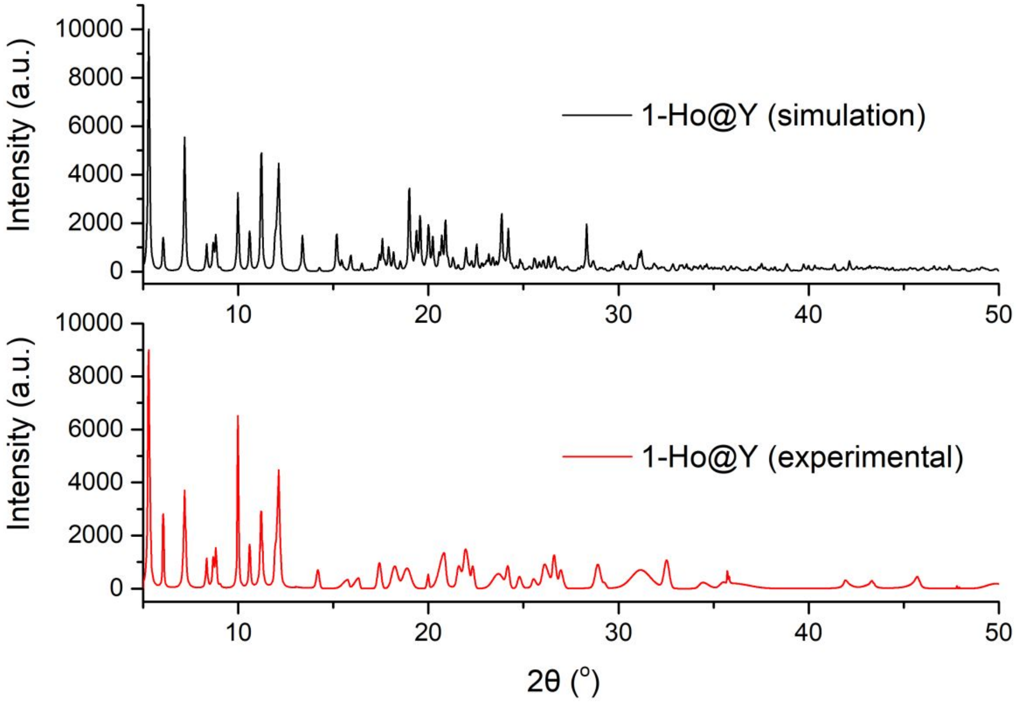

Figure S8. Experimental and simulated powder X-ray diffraction (p-XRD) patterns of **1-Ho@Y**. The simulated pattern is calculated based on the structural model from the single-crystal X-ray diffraction data.

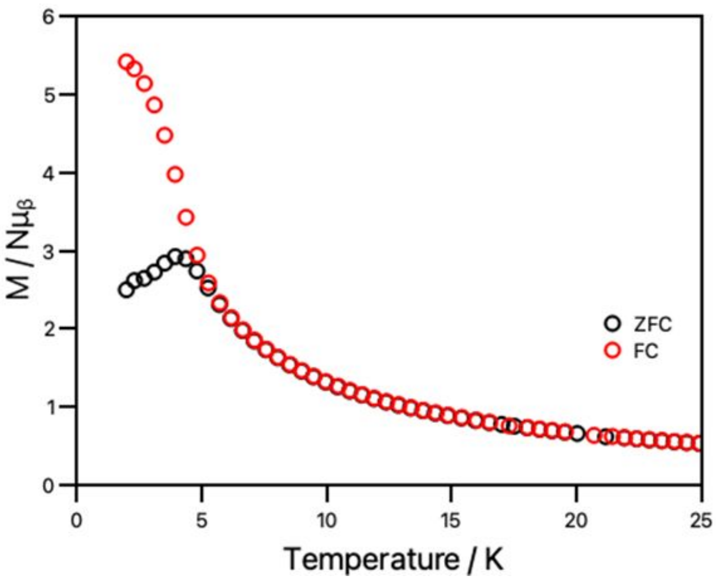

Figure S9. Variable-temperature magnetization of complex **1-Dy**, under field-cooled (FC) and zero-field-cooled (ZFC) conditions with an applied field of 1000 Oe.

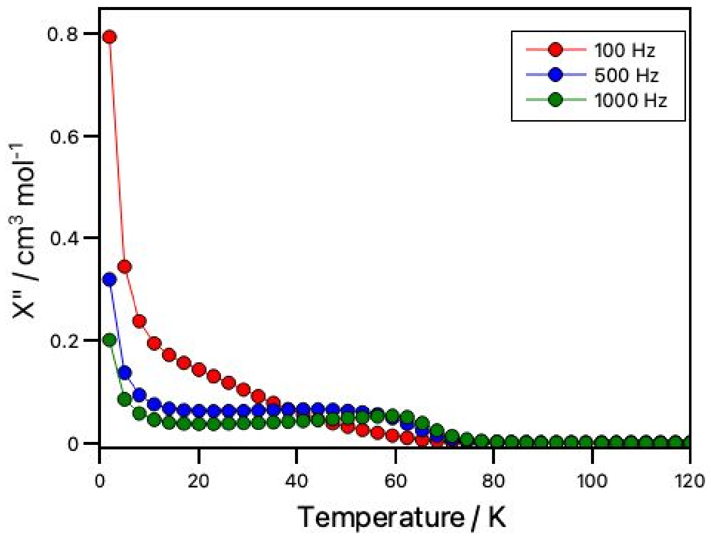

Figure S10. Temperature dependence of the out-of-phase (χ_M'') ac magnetic susceptibility at zero external dc field for **1-Dy**, measured in a 3.0 G ac field oscillating at the indicated frequencies. The solid lines are guides only.

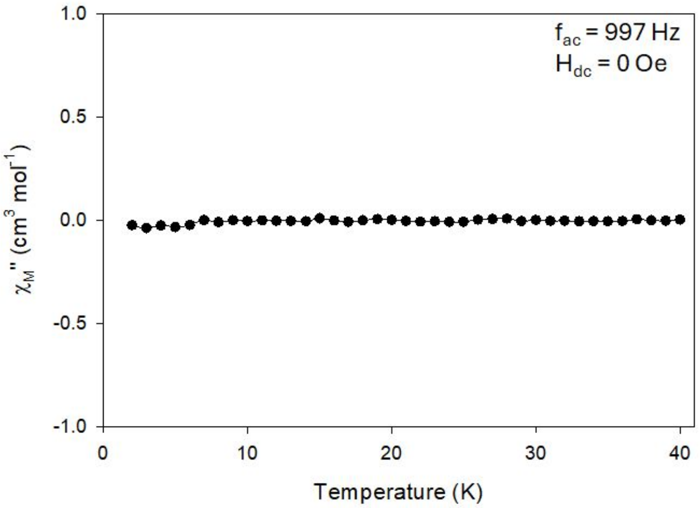

Figure S11. Temperature dependence of the out-of-phase ac susceptibility at a maximum frequency of 997 Hz, under zero applied dc field for **1-Ho**.

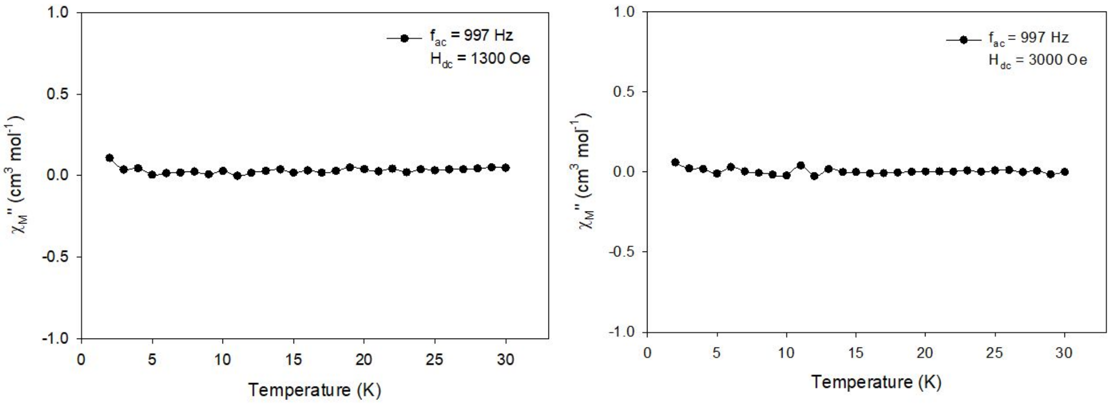

Figure S12. Temperature dependence of the out-of-phase ac susceptibility at maximum frequency of 997 Hz, under external dc fields of 1300 Oe (left) and 3000 Oe (right), for **1-Ho**.

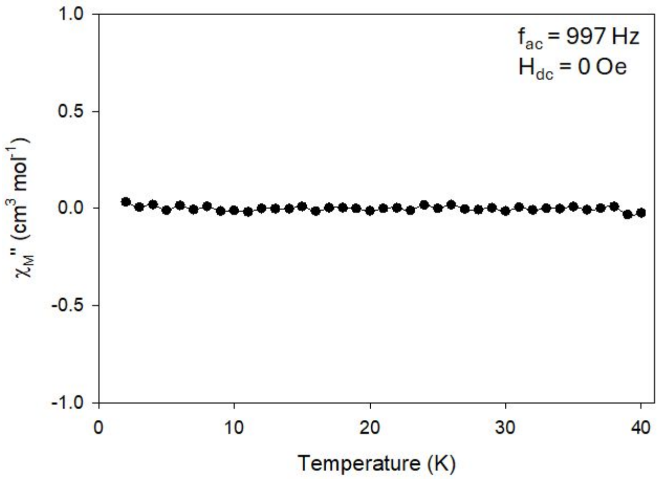

Figure S13. Temperature dependence of the out-of-phase ac susceptibility at a maximum frequency of 997 Hz, under zero applied dc field for **1-Ho@Y**.

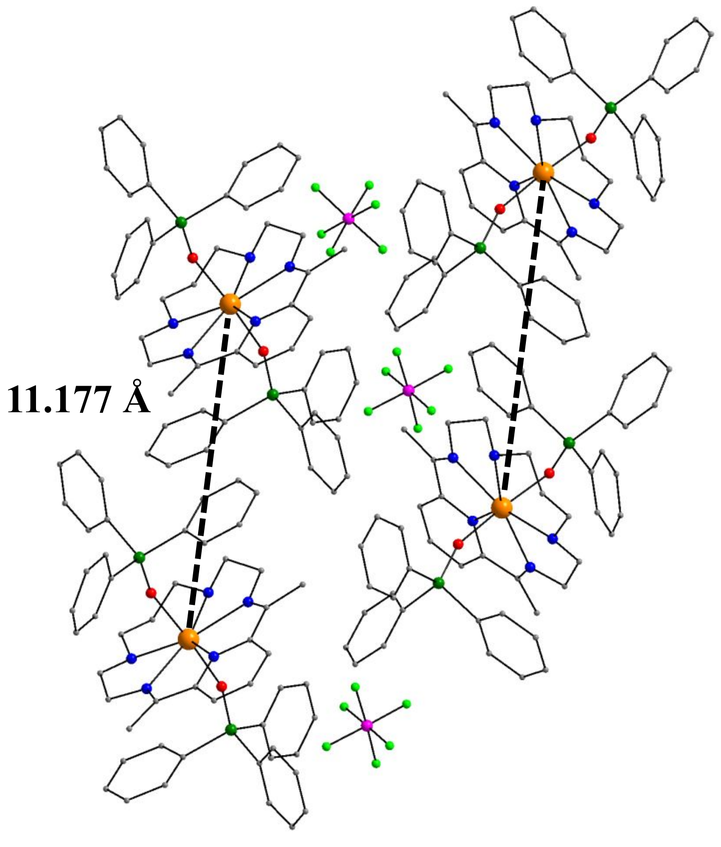

Figure S14. A portion of the repeating monomeric complexes in the crystal of **1-Ho@Y** viewed along the *c*-axis and visualization of the shortest intermetallic distance as black dashed lines. Color scheme: Y^{III}/Ho^{III}, orange; O, red; N, blue; C, grey; Si, olive; P, magenta; F, green. H-atoms are omitted for clarity.

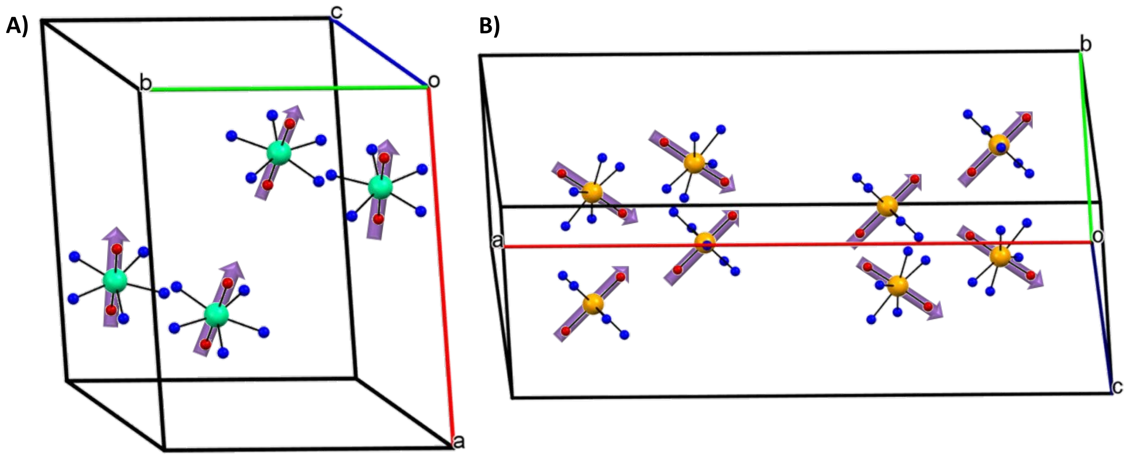

Figure S15. Alignments of the O_{ax}-Ho-O_{ax} and O_{ax}-Y-O_{ax} molecular *z*-axes between neighboring coordination compounds in the crystals of **1-Ho** (A) and **1-Y** (B), respectively. To emphasize the angles between the O_{ax}-Ln-O_{ax} axes of neighboring compounds, most atoms have been omitted for clarity.

3. Ab initio calculations

Table S8. Computed energy levels (the ground state is set at zero) composition of the *g*-tensor (g_x, g_y, g_z) and the main components (>10%) of the wavefunction for each m_J state of the ground-state multiplet $^6H_{15/2}$ for **1-Dy**, at the CASSCF level.

Energy (cm^{-1})	g_x	g_y	g_z	Wavefunction
0	0.0003	0.0007	17.8963	99.9% |±15/2>
398.7	0.0963	0.1080	16.9432	97.8% |±13/2>
629.1	0.8216	2.2628	15.0462	58.8%|±1/2>+18.6%|±3/2>+17.8 % |±11/2>
690.2	7.6419	6.7393	1.9718	53.0%|±11/2>+28.6% |±1/2>
724.6	9.7908	5.5318	0.1434	25.0%|±11/2>+54.2%|±3/2>
791.6	7.3681	6.2675	2.4884	15.5%|±9/2>+68.7%|±5/2>
854.5	2.2765	3.9862	14.0076	55.8%|±9/2>+31.6%|±7/2>
891.0	0.1104	2.0660	16.2768	23.8%|±9/2>+57.7%|±7/2>+11.7 %|±5/2>

Table S9: CASSCF calculated average transition magnetic moment matrix elements in units μ^2 for **1-Dy**.

**Multiplet**	**Matrix Elements**	**Average**	**Multiplet**	**Matrix Elements**	**Average**
	+I* to *-I			***I* to *I*+3**	
1	$\langle +1.1 \vert -1.1$	1.64261E-4	1	$\langle +1.1 \vert +4.1$	0.06474
2	$\langle +2.1 \vert -2.1$	0.0341	1	$\langle +1.1 \vert -4.1$	0.03708
3	$\langle +3.1 \vert -3.1$	2.72184	2	$\langle +2.1 \vert +5.1$	1.05298
4	$\langle +4.1 \vert -4.1$	1.54977	2	$\langle +2.1 \vert -5.1$	0.08017
5	$\langle +5.1 \vert -5.1$	2.13923	3	$\langle +3.1 \vert +6.1$	0.79911
6	$\langle +6.1 \vert -6.1$	1.75178	3	$\langle +3.1 \vert -6.1$	0.25335
7	$\langle +7.1 \vert -7.1$	1.46038	4	$\langle +4.1 \vert +7.1$	1.62155
8	$\langle +8.1 \vert -8.1$	0.79701	4	$\langle +4.1 \vert -7.1$	0.38994
	***I* to *I*+1**		5	$\langle +5.1 \vert +8.1$	0.59888
1	$\langle +1.1 \vert +2.1$	1.76289	5	$\langle +5.1 \vert -8.1$	0.67621
1	$\langle +1.1 \vert -2.1$	7.51456E-4		***I* to *I*+4**	
2	$\langle +2.1 \vert +3.1$	1.29879	1	$\langle +1.1 \vert +5.1$	0.05032
2	$\langle +2.1 \vert -3.1$	0.17911	1	$\langle +1.1 \vert -5.1$	0.02653
3	$\langle +3.1 \vert +4.1$	1.3848	2	$\langle +2.1 \vert +6.1$	0.16801
3	$\langle +3.1 \vert -4.1$	1.78779	2	$\langle +2.1 \vert -6.1$	0.15405
4	$\langle +4.1 \vert +5.1$	0.89458	3	$\langle +3.1 \vert +7.1$	0.58472
4	$\langle +4.1 \vert -5.1$	1.55868	3	$\langle +3.1 \vert -7.1$	0.27872
5	$\langle +5.1 \vert +6.1$	2.43982	4	$\langle +4.1 \vert +8.1$	0.98488
5	$\langle +5.1 \vert -6.1$	0.62813	4	$\langle +4.1 \vert -8.1$	0.4092
6	$\langle +6.1 \vert +7.1$	1.74924		***I* to *I*+5**	
6	$\langle +6.1 \vert -7.1$	1.6614	1	$\langle +1.1 \vert +6.1$	0.05336
7	$\langle +7.1 \vert +8.1$	1.54091	1	$\langle +1.1 \vert -6.1$	0.00886

7	$\langle +7.1 \| -8.1$	1.49608	2	$\langle +2.1 \| +7.1$	0.16717
	***I* to *I*+2**		2	$\langle +2.1 \| -7.1$	0.1695
1	$\langle +1.1 \| +3.1$	0.03719	3	$\langle +3.1 \| +8.1$	0.34713
1	$\langle +1.1 \| -3.1$	0.06595	3	$\langle +3.1 \| -8.1$	0.15069
2	$\langle +2.1 \| +4.1$	1.72916		***I* to *I*+6**	
2	$\langle +2.1 \| -4.1$	0.10279	1	$\langle +1.1 \| +7.1$	0.06724
3	$\langle +3.1 \| +5.1$	0.67613	1	$\langle +1.1 \| -7.1$	0.02987
3	$\langle +3.1 \| -5.1$	1.88735	2	$\langle +2.1 \| +8.1$	0.24979
4	$\langle +4.1 \| +6.1$	1.77041	2	$\langle +2.1 \| -8.1$	0.05779
4	$\langle +4.1 \| -6.1$	0.50596		***I* to *I*+7**	
5	$\langle +5.1 \| +7.1$	1.32255	1	$\langle +1.1 \| -8.1$	0.06524
5	$\langle +5.1 \| -7.1$	0.50564	1	$\langle +1.1 \| +8.1$	0.01646
6	$\langle +6.1 \| +8.1$	1.80326			
6	$\langle +6.1 \| -8.1$	0.63874			

Table S10. Computed energy levels (the ground state is set at zero) composition of the g-tensor (g_x, g_y, g_z) and the main components (>10%) of the wavefunction for each m_J state of the ground-state multiplet $^5I_8$ for **1-Ho**, at the CASSCF level.

**Energy (cm^{-1})**	g_x	g_y	g_z	Δ_{tun} **(cm^{-1})**
0.0000 / 0.1485	0.0000	0.0000	19.8497	0.15
298.3 / 303.5	0.0000	0.0000	15.7435	5.2
317.2 / 326.0	0.0000	0.0000	9.1271	8.8
334.0 / 347.1	0.0000	0.0000	6.4945	13.1
358.6 / 371.3	0.0000	0.0000	6.9916	12.7
388.0 / 400.6	0.0000	0.0000	13.9512	12.6
427.9 / 432.5	0.0000	0.0000	8.6295	4.6
439.3 / 455.7	0.0000	0.0000	6.9957	16.4

Table S11. Computed energy levels (the ground state is set at zero) composition of the wavefunction for each m_J state of the ground-state multiplet $^5I_8$ for **Ho**, at the CASSCF level.

E / cm^{-1}	0	0.1491	298.3	303.5	317.2	326	334	347.1	358.6	371.3	388	400.6	427.9	432.5	439.3	455.7
|-8>	49.8	49.8	0.0	0.0	0.0	0.0	0.1	0.0	0.0	0.0	0.1	0.0	0.0	0.0	0.0	0.0
|-7>	0.0	0.0	5.0	0.6	23.4	21.4	0.4	0.1	19.0	21.9	2.2	4.8	0.4	0.5	0.0	0.2
|-6>	0.0	0.0	0.1	0.1	0.3	0.3	0.6	7.5	1.8	0.9	3.9	1.2	10.8	31.0	23.5	5.1
|-5>	0.0	0.0	0.6	0.4	1.4	3.3	0.5	3.9	8.1	5.0	3.4	20.0	25.7	9.5	1.0	11.5
|-4>	0.1	0.1	0.8	9.1	0.9	1.3	26.9	0.1	0.8	5.1	31.2	0.2	1.8	1.6	7.4	6.1
|-3>	0.0	0.0	35.8	1.3	10.2	2.8	0.5	0.1	1.2	7.1	0.6	22.2	1.3	1.8	0.0	8.0
|-2>	0.0	0.0	0.7	35.0	0.1	2.9	17.3	5.8	0.7	2.7	4.3	0.7	1.1	3.0	17.4	4.4
|-1>	0.0	0.0	6.7	1.0	13.0	17.8	2.1	5.5	14.0	7.2	4.1	0.3	8.9	2.6	0.2	10.1
|0>	0.0	0.0	0.6	5.0	1.2	0.2	3.3	54.1	9.0	0.1	0.3	1.4	0.1	0.1	1.1	9.3
|+1>	0.0	0.0	6.7	1.0	13.0	17.8	2.1	5.5	14.0	7.2	4.1	0.3	8.9	2.6	0.2	10.1
|+2>	0.0	0.0	0.7	35.0	0.1	2.9	17.3	5.8	0.7	2.7	4.3	0.7	1.1	3.0	17.4	4.4
|+3>	0.0	0.0	35.8	1.3	10.2	2.8	0.5	0.1	1.2	7.1	0.6	22.2	1.3	1.8	0.0	8.0
|+4>	0.1	0.1	0.8	9.1	0.9	1.3	26.9	0.1	0.8	5.1	31.2	0.2	1.8	1.6	7.4	6.1
|+5>	0.0	0.0	0.6	0.4	1.4	3.3	0.5	3.9	8.1	5.0	3.4	20.0	25.7	9.5	1.0	11.5
|+6>	0.0	0.0	0.1	0.1	0.3	0.3	0.6	7.5	1.8	0.9	3.9	1.2	10.8	31.0	23.5	5.1
|+7>	0.0	0.0	5.0	0.6	23.4	21.4	0.4	0.1	19.0	21.9	2.2	4.8	0.4	0.5	0.0	0.2
|+8>	49.8	49.8	0.0	0.0	0.0	0.0	0.1	0.0	0.0	0.0	0.1	0.0	0.0	0.0	0.0	0.0

Table S12. Crystal field Hamiltonian, given as $\hat{H}_{CF} = \sum_{k,q} B_k^q O_k^q$, and the extended Stevens operator coefficients B_k^q extracted from CASSCF calculations for compounds **1-Dy** and **1-Ho**.

		B_k^q	
k	*q*	**1-Dy**	**1-Ho**
2	-2	-0.18355	0.06255
2	-1	-0.31354	0.11954
**2**	**0**	**-3.88965**	**-1.23166**
2	1	-0.51035	0.01683
2	2	1.03805	-0.36144
4	-4	0.00776	0.00287
4	-3	-0.00946	-0.00145
4	-2	-3.85681E-4	3.66848E-4

4	-1	1.34621E-4	-0.00199
**4**	**0**	**-0.01494**	**-0.00646**
4	1	0.00199	0.00106
4	2	0.00149	-8.38289E-4
4	3	-0.00133	0.00423
4	4	0.01072	0.00526
6	-6	-2.03197E-4	-2.13429E-4
6	-5	-1.23429E-4	1.57615E-5
6	-4	8.20571E-5	-6.74383E-5
6	-3	-4.17944E-5	-1.82856E-5
6	-2	1.05889E-5	1.05775E-5
6	-1	1.7564E-5	2.24955E-5
**6**	**0**	**3.51252E-5**	**-2.94806E-5**
6	1	2.71915E-5	-6.63601E-6
6	2	-1.95239E-6	9.40265E-6
6	3	1.72105E-5	-5.82059E-5
6	4	1.01904E-4	-1.09979E-4
6	5	5.03325E-5	1.62385E-4
6	6	-6.54068E-5	-1.22452E-4

References:

(S1) Bastida, R.; de Blas, A.; Castro, P.; Fenton, D. E.; Macías, A.; Rial, R.; Rodríguez, A.; Rodríguez-Blas, T. Complexes of Lanthanide Ions with Macrocyclic Ligands Containing Pyridine Head Units, *J. Chem. Soc., Dalton Trans.*, **1996**, 1493-1497.

(S2) Coates, J. Interpretation of Infrared Spectra, A Practical Approach. In *Encyclopedia of Analytical Chemistry*; Meyers, R. A., Ed.; Wiley, 2000.

(S3) Heyns, A. M. The i.r. and Raman Spectra of Sodium Hexafluorophosphate Monohydrate, $NaPF_6·H_2O$, *Spectrochim. Acta - A: Mol. Biomol. Spectrosc.*, **1977**, *33*, 315-322.

(S4) (a) Hirata, T. Evolution of the Infra-Red Vibrational Modes upon Thermal Oxidation of Si Single Crystals. *J. Phys. Chem. Solids*, **1997**, *58*, 1497–1501. (b) Warring, S. L.; Beattie, D. A.; McQuillan, A. J. Surficial Siloxane-to-Silanol Interconversion during Room-Temperature Hydration/Dehydration of Amorphous Silica Films Observed by ATR-IR and TIR-Raman Spectroscopy. *Langmuir* **2016**, *32*, 1568–1576.
